# Seascape genetics of the spiny lobster *Panulirus homarus* in the Western Indian Ocean: Understanding how oceanographic features shape the genetic structure of species with high larval dispersal potential

**DOI:** 10.1002/ece3.4684

**Published:** 2018-11-16

**Authors:** Sohana P. Singh, Johan C. Groeneveld, Michael G. Hart‐Davis, Björn C. Backeberg, Sandi Willows‐Munro

**Affiliations:** ^1^ Oceanographic Research Institute Marine Parade South Africa; ^2^ School of Life Sciences University of KwaZulu‐Natal Pietermaritzburg South Africa; ^3^ Institute for Coastal and Marine Research Nelson Mandela University Port Elizabeth South Africa; ^4^ Department of Oceanography, Nansen‐Tutu Centre for Marine Environmental Research University of Cape Town South Africa; ^5^ Egagasini Node South African Environmental Observation Network Cape Town South Africa; ^6^ Council for Scientific and Industrial Research, Natural Resources and the Environment Coastal Systems Research Group Stellenbosch South Africa; ^7^ Nansen Environmental and Remote Sensing Center Bergen Norway

**Keywords:** contact zone, larval retention, marine biogeography, ocean circulation, Phylogeography

## Abstract

This study examines the fine‐scale population genetic structure and phylogeography of the spiny lobster *Panulirus homarus* in the Western Indian Ocean. A seascape genetics approach was used to relate the observed genetic structure based on 21 microsatellite loci to ocean circulation patterns, and to determine the influence of latitude, sea surface temperature (SST), and ocean turbidity (KD490) on population‐level processes. At a geospatial level, the genetic clusters recovered corresponded to three putative subspecies, *P. h. rubellus* from the SW Indian Ocean, *P. h. megasculptus* from the NW Indian Ocean, and *P. h. homarus* from the tropical region in‐between. Virtual passive Lagrangian particles advected using satellite‐derived ocean surface currents were used to simulate larval dispersal. In the SW Indian Ocean, the dispersion of particles tracked over a 4‐month period provided insight into a steep genetic gradient observed at the Delagoa Bight, which separates *P. h. rubellus* and *P. h. homarus*. South of the contact zone, particles were advected southwestwards by prevailing boundary currents or were retained in nearshore eddies close to release locations. Some particles released in southeast Madagascar dispersed across the Mozambique Channel and reached the African shelf. Dispersal was characterized by high seasonal and inter‐annual variability, and a large proportion of particles were dispersed far offshore and presumably lost. In the NW Indian Ocean, particles were retained within the Arabian Sea. Larval retention and self‐recruitment in the Arabian Sea could explain the recent genetic divergence between *P. h. megasculptus* and *P. h. homarus*. Geographic distance and minimum SST were significantly associated with genetic differentiation in multivariate analysis, suggesting that larval tolerance to SST plays a role in shaping the population structure of *P. homarus*.

## 1 INTRODUCTION

Seascape genetics, where population genetics draws on ecology, oceanography, and geography to explain connectivity and spatial genetic structure, was recently reviewed by Selkoe, D’Aloia, et al. (2016). Seascape genetics provides a conceptual framework for understanding the multiple historical and contemporary processes that shape dispersal and gene flow in the sea (Portnoy et al., 2014; Riginos, Douglas, Jin, Shanahan, and Treml, 2011; Selkoe, Henzler, & Gaines, 2008). For example, larvae that are adapted to drifting in the water column may rely on ocean currents, eddies, and fronts for dispersal, but can also use larval positioning or swimming behavior to remain in favorable habitats or to move toward the coast (Schmalenbach and Buchholz 2010; Butler, Paris, Goldstein, Matsuda, & Cowen, 2011). Ocean circulation can actively promote genetic exchange over long distances (Chiswell, Wilkin, Booth, & Stanton, 2003; Groeneveld, von der Heyden, & Matthee, 2012) or constitute a barrier to gene flow, even when populations are geographically close to each other (Ayre & Dufty, 1994; Palumbi, 1994; Teske et al., 2008). Common seascape correlations of spatial genetic metrics include geography, temperature, ocean transport, habitat patch size or continuity, chlorophyll a, or the presence of biogeographic breaks/ecoregions (reviewed in Selkoe, D’Aloia, et al., 2016). Environmental gradients act on dispersal processes and gene flow through influencing larval survival and post‐settlement fitness—and hence recruitment success (Nanninga, Saenz‐Agudelo, Manica, & Berumen, 2013; Robitzch, Banguera‐Hinestroza, Sawall, Al‐Sofyani, & Voolstra, 2015).

The scalloped spiny lobster *Panulirus homarus* is widespread in the Indo‐West Pacific, extending northwards from southeastern Africa to the Arabian Sea, and eastwards to Japan, Indonesia, and northern Australia (Holthuis, 1991). *Panulirus homarus* comprises several putative subspecies, and its evolutionary history and phylogeography have been popular subjects in the recent literature (Farhadi, Farahmand, Nematollahi, Jeffs, & Lavery, 2013; Farhadi et al., 2017, Lavery et al., 2014; Reddy, MacDonald, Groeneveld, & Schleyer, 2014; Singh, Groeneveld, Al‐Marzouqi, & Willows‐Munro, 2017). Analyses of mitochondrial and nuclear sequences from several genetic markers and microsatellite loci have come to broadly similar conclusions, that at least three or four separate evolutionary lineages exist, occupying distinct geographic ranges. The genetic information largely agrees with morphological groupings (Berry, 1974a). *Panulirus homarus rubellus* (red megasculpta form) from the Southwest (SW) Indian Ocean is the most divergent and possibly a separate species based on phylogenetic analyses using mitochondrial and nuclear markers (Farhadi et al., 2017; Lavery et al., 2014; Singh et al., 2017). *Panulirus homarus* “brown” is restricted to the Marquesas Islands in the Central Pacific, and *P. homarus homarus* (green microsculpta form) is widespread in intervening areas (Farhadi et al., 2017). The status of *P. homarus megasculptus* (green megasculpta form) from the Northwest (NW) Indian Ocean (mainly Arabian Sea) remains unclear. Neither Singh et al. (2017) nor Lavery et al. (2014) could find a phylogenetic basis for distinguishing it from *P. h. homarus* from the SW Indian Ocean based on mtDNA and nuclear loci, but Farhadi et al. (2013), Farhadi et al. (2017) found evidence for reduced gene flow between the NW Indian Ocean and populations in Kenya and Tanzania, further to the south.

**Figure 1:**
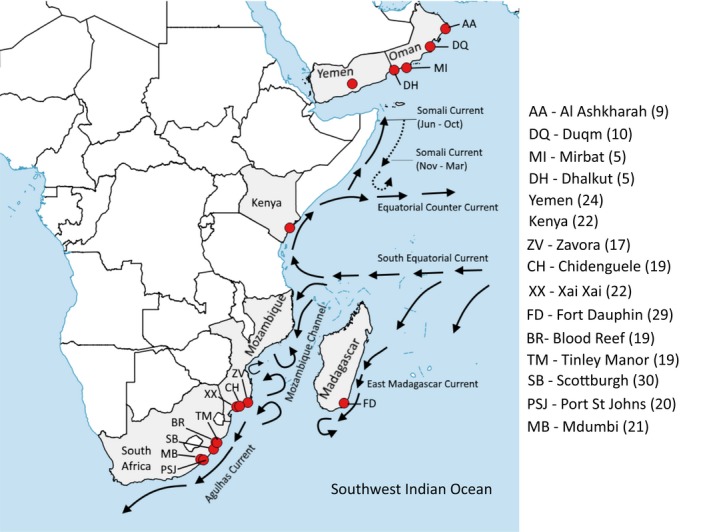
Map of *Panulirus homarus* sampling locations and the number of samples collected per locality. The major current systems (adapted from Lutjeharms, 2006) are also shown

The Western Indian Ocean extends from eastern South Africa to the Arabian Gulf, including waters around Madagascar and several small island states, such as Mauritius, Seychelles, and Comoros (Figure 1). It is a tropical/subtropical region with high biodiversity and endemism, brought about by diverse habitats along the continental shelf, and around isolated islands or subsurface plateaus (van der Elst et al. 2009; Halo, Malauene, & Ostrowski, 2017). Two western boundary currents dominate the coastal oceanography: the Agulhas Current to the south and the Somali Current to the north. The Somali Current reverses its flow direction seasonally, under the influence of the SW monsoon (flows northwards) and the northeast (NE) monsoon (flows southwards) seasons (Schott & McCreary, 2001). It also receives waters from the East African Coastal Current along coastal Tanzania and Kenya. Water from the South Equatorial Current enters the northern Mozambique Channel, forming a series of eddies which propagate southwards through the channel (Halo et al., 2017; Otwoma & Kochzius, 2016). These waters are augmented by eddies originating from the East Madagascar Current at the southern end of the channel (de Ruijter et al., 2004; Ridderinkhof, Bars, Heydt, & Ruijter, 2013), and their confluence forms the upper Agulhas Current off southern Mozambique and eastern South Africa.

Spalding et al. (2007) subdivided the Western Indian Ocean into 12 marine ecoregions, or “areas of relatively homogenous species composition, clearly distinct from adjacent systems.” Biogeographic forcing agents characteristic of ecoregions include upwelling cells (e.g., the Central Somali Coast), nutrient inputs from freshwater influx (Sofala Bight/Swamp Coast), the influence of ocean currents (Northern Monsoon Coastal Current), bathymetric or coastal complexity (East African Coral Coast), or differences in temperature regimes or sediments. Toward the south, the subtropical Natal ecoregion is characterized by high species diversity, resulting from an influx of species from the Indo‐Pacific, transported by the Agulhas Current (Bustamante & Branch 1996; Spalding et al., 2007). Madagascar comprises two separate ecoregions, Southeast Madagascar with cooler water and upwelling cells, and Western and Northern Madagascar where this upwelling is negligible or absent (Spalding et al., 2007).

The concordance between marine biogeographic and phylogeographic boundaries is increasingly recognized in the literature (Bowen et al., 2016; Teske, Froneman, Barker, & McQuaid, 2007; Teske, McQuaid, Froneman, & Barker, 2006; Teske, Winker, McQuaid, & Barker, 2009). Where individual species are distributed across two or more biogeographic provinces, shifts in genotype frequencies often align with biogeographic boundaries, providing intraspecific concordance between biogeography and phylogeography. Recent examples among marine taxa include sea snails (Brante, Fernández, & Viard, 2012); clams (DeBoer et al.., 2014); Indo‐Pacific goatfish, *Mulloidichthys flavolineatus* (Fernandez‐Silva et al., 2015); spiny lobster, *Panulirus penicillatus* (Iacchei, Gaither, Bowen, & Toonen, 2016); barnacles (Ewers‐Saucedo et al., 2016); and seaweeds in the southeast Pacific (Guillemin et al., 2016).

We used biological information, habitats, and ocean surface currents in a seascape genetics approach to explore the genetic structure observed within the *P. homarus* subspecies complex in the Western Indian Ocean. The hypothesis that isolation by distance is the main driver of differentiation among subspecies was tested. Alternatively, biogeographic breaks determined by ocean circulation patterns and environmental gradients may have constrained gene flow, resulting in genetic differentiation between the three groups. Specific aims were to: (a) evaluate potential phylogeographic boundary zones; (b) use virtual particle tracking simulations to infer larval dispersal patterns based on surface currents; and (c) determine the extent to which genetic structure correlates with environmental variables and simulated larval movement.

## 2 MATERIALS AND METHODS

### 2.1 Microsatellite sampling

A total of 271 *Panulirus homarus* specimens were collected from 15 sites in the Western Indian Ocean: five sites from eastern South Africa, three from Mozambique, one each in southern Madagascar, Kenya and Yemen, and four in Oman (Figure 1). All specimens were identified to subspecies level based on phenotypic and geographic information. There were 43 individuals in the *P. h. homarus* group (22 from Kenya; 20 from Mozambique; 1 from South Africa), 53 in the *P. h. megasculptus* group (29 from Oman; 24 from Yemen), and 175 in the *P. h. rubellus* group (38 from Mozambique; 29 from Madagascar; and 108 from South Africa). DNA was extracted from pereiopod tissue using the Zymo gDNA Universal DNA extraction kit (Inqaba, Zymo), as per the manufacturers protocol.

A panel of 21 microsatellite loci, consisting of eight loci (Orn4, Orn5, Orn11, Orn12, Orn16, Orn17, Orn21, and Orn32) described by Dao, Todd, and Jerry (2013) and 13 loci (Pho G01, Pho G03, Pho G21, Pho G22, Pho G25, Pho G27, Pho G30, Pho G32, Pho G35, Pho G36, Pho G42, Pho G53, and Pho G58) described by Delghandi et al. (2015), was chosen for PCR amplification. Forward primers were labeled with a fluorescent dye on the 5′ end. The microsatellite panel was partitioned into six multiplex reactions and a single reaction for Orn 17 (Supporting Information Table S1).

PCR products were carried out in 10 µl volume reactions, which contained 5 µl of 1× Kapa 2G Fast Multiplex Mastermix (Kapa Biosystems), 0.2 µmol/L of each primer, and 1 µl of 10–50 ng of genomic DNA. For multiplexes A, B, C, and Orn 17, cycling conditions were as follows: an initial denaturation at 95°C for 5 min, followed by 28 cycles of denaturation at 95°C for 30 s, annealing at 60°C for 90 s, extension at 72°C for 30 s, and a final extension step at 60°C for 30 min. For multiplexes E, F, and G, cycling conditions were as follows: an initial denaturation step at 95°C for 15 min, 32 cycles of denaturation at 94°C for 30 s, annealing at 60°C for 90 s, extension for 72°C for 60 s, and a final extension at 72°C for 30 min.

Fragment analysis of amplified products was done at Central Analytical Facility at Stellenbosch University, South Africa. GeneMarker v. 2.4.0 (Soft Genetics) was used to score the genotypes. To monitor the consistency in genotype scoring, 20% of the samples were re‐amplified and scored. Individuals with <20% missing data were considered for further analyses. Microchecker v.2.2.3 (van Oosterhout, Hutchinson, Wills, & Shipley, 2004) was used to check for genotyping errors such as allelic dropout, stuttering, and the presence of null alleles. The Expectation Maximization Algorithm (EM) implemented in FreeNA (Chapuis & Estoup, 2007) was used to estimate the null allele frequencies for each marker and population. Bootstrap resampling over loci was conducted with 100,000 replicates. The excluding null alleles (ENA) method was used to compare null allele corrected and uncorrected global and population *F*_ST_ values using paired *t* tests.

### 2.2 Genetic diversity indices

GenAlex v.6.5 (Peakall & Smouse, 2006) was used to compute the number of alleles per locus and population. Arlequin v. 3.5.2.2 (Excoffier & Lischer, 2010) was used to calculate observed and expected heterozygosities and to test for departures from Hardy–Weinberg Equilibrium (HWE) and linkage equilibrium (10,000 replicates). The Bonferroni correction was applied for multiple comparisons. The allelic richness per locus was calculated using FSTAT v.2.9.3.2 (Goudet, 1995). The polymorphic information content (PIC) for each locus was estimated using Cervus v.3.0.7 (Kalinowski, Taper, & Marshall, 2007; Marshall, Slate, Kruuk, & Pemberton, 1998; Slate, Marshall, & Pemberton, 2000).

### 2.3 Genetic structure and gene flow

Population genetic structure between the three subspecies and between different localities was evaluated using STRUCTURE v. 2.3.4 (Pritchard, Stephens, & Donnelly, 2000). *K* was set from 1 to 12. Each *K* was run for 10 iterations. The admixture model was chosen and run with and without sampling location as a prior. A total of 1,000,000 MCMC iterations were performed after a burn‐in period of 250,000 iterations. Structure Harvester (Earl & von Holdt, 2012; https://taylor0.biology.ucla.edu/structureHarvester/) and Pophelper (Francis, 2017; https://pophelper.com/) were used to identify the number of genetic clusters (*K*) that fit the data best (Evanno, Regnaut, & Goudet, 2005). An analysis of molecular variance (AMOVA) was performed using Arlequin, to assess the magnitude of genetic differentiation between the groups identified by the STRUCTURE analysis.

The role of seascape features in population genetic structure, and potential contact zones, was inferred using the Geneland v.4.0.3 R‐package (Guillot, Mortier, & Estoup, 2005). This method couples multilocus microsatellite genotype data and geospatial information by using geographic sampling locality co‐ordinates as priors, enabling the detection of subtle population structure, and also to infer the locations of genetic discontinuities or transition zones (Guillot et al., 2005). The analysis consisted of 10 independent runs of 1,000,000 MCMC iterations, sampling every 100 generations. The number of predefined genetic clusters (*K*) ranged from 1 to 12. The spatial model was employed, with and without accounting for null alleles. The correlated allele frequencies model was chosen to detect subtle genetic structure (Guillot, 2008).

The null hypothesis of isolation by distance was examined by testing the relationship between genetic and geographic distance using a Mantel test. The analysis was carried out using the program IBDWS (Jensen, Bohonak, & Kelley, 2005, https://ibdws.sdsu.edu/~ibdws/). The genetic distances (*F*_ST_/(1 – *F*_ST_) were regressed against the geographic distances (straight‐line distances on Google Earth) with 1,000 randomizations between populations.

### 2.4 Environmental factors

The relationship between genetic differentiation of the *P. homarus* groups (pairwise *F*_ST_), geography, and environment was tested using the multivariate multiple regression approach of a distance‐based redundancy analysis (dbRDA) (Legendre & Anderson, 1999). Monthly composite oceanic variable data of ocean productivity chlorophyll *a* (chl *a*, mg/m^3^), sea surface temperature (mean, maximum and minimum SST, °C), and turbidity (diffuse attenuation coefficient KD490; m^−1^) were obtained from the GMIS Marine Geodatabase (mcc.jrc.ec.europa.eu), based on the NASA MODIS terra satellite at a 4‐km spatial resolution. Data were collected from 2012 to 2015, coinciding with the genetic sampling regime. A matrix of genetic differentiation between sampling sites (*F*_ST_) was used as the response variable, and mean measurements of environmental variables (chl *a*, mean, max and min SST, turbidity), and larval recruits (based on estimates from ocean modeling simulations as the number of larvae that reach settlement at each site on the coast) were used as the explanatory variables. The dbRDA analysis was carried out using the “capscale” function in the Vegan package (Oksanen et al., 2015) in R. The correlation among variables was measured using Pearson's correlation coefficient in Vegan. Where variable pairs were highly correlated (Rellstab, Gugerli, Eckert, Hancock, & Holderegger, 2015), the biologically more relevant one to *P. homarus* was used. Minimum SST was considered an important correlate because it decreases along a gradient from tropical (*P. h. homarus* preference) to subtropical conditions (*P. h. rubellus* preference) (Berry, 1971b, 1974a ). SST is expected to affect phyllosoma larvae that disperse in the upper layers of the water column. Turbidity was selected because *P. homarus* juveniles and adults prefer turbid conditions in the shallow subtidal zone (Berry, 1971b; Holthuis, 1991). The Euclidean geographic distance matrix was transformed into a continuous rectangular vector by principal co‐ordinates analysis (PCoA) using the “pcnm” function in Vegan. The “ordistep” function was used to identify the most significant variables to include in the model (Supporting Information Table S2). The “varpart” function in Vegan was used to estimate the amount of genetic differentiation due to each predictor variable. Significance of the full model was assessed by comparing it to the null model containing only the intercept, and significance of each predictor variable was tested by comparing models which test each predictor after removing the effects of the other predictors. Significance tests were carried out using the “ANOVA.cca” function in Vegan.

### 2.5 Larval dispersal simulations

We explored the effects of ocean circulation on larval dispersal and settlement, and hence genetic differentiation, in the virtual particle tracking tool, Parcels v.0.9 (Probably A Really Computationally Efficient Lagrangian Simulator; Lange & van Sebille, 2017). Lagrangian particle trajectories were simulated using Parcels with the equation:

$$X\left(t+\Delta t\right)=X\left(t\right)+\int_{t}^{t+\Delta t}v(x,\tau )d\tau$$

where X is the three‐dimensional position of a particle and $v\left(x,\tau \right)$ is the three‐dimensional velocity field at that location from an ocean general circulation model.

Ocean velocity data were obtained from the GlobCurrent Project (Johannessen et al., 2016; https://www.globcurrent.org/) which provides daily average surface ocean current estimates for the world's oceans at 25‐km spatial resolution by merging data from multiple satellite altimeter missions with gravity models and combining these with Ekman currents (at surface and 15 m depth) estimated from drogued surface drifter data, Argo floats, and near‐surface winds.

The u‐ and v‐component geostrophic velocities are defined by the following equations (Saraceno, Strub, & Kosro, 2008):

$$\upsilon_{\mathrm{geo}}=-\left(\frac{g}{f}\right).\frac{\delta \eta_{y\left[0\right]}-\delta \eta_{y\left[1\right]}}{\delta y}$$

$$\nu_{\mathrm{geo}}=\left(\frac{g}{f}\right).\frac{\delta \eta_{x\left[0\right]}-\delta \eta_{x\left[1\right]}}{\delta x}$$

where *υ* represents the zonal component, *ν* is the meridional component, δη is the variation in the sea surface height, δx/δy is the distance between grid points, *g* is the gravitational acceleration, and *f* is the Coriolis force.

These products are combined with the Ekman currents estimated from Argo floats and drifter data in conjunction with wind stress estimates from the European Centre for Medium‐Range Weather Forecasts (ECMWF). At depth z (0 and 15 m), the Ekman response (${\vec{\upsilon }}_{\mathrm{ek}})$to the wind stress ($\vec{\tau }$) is expressed by Rio, Mulet, and Picot (2014):${\vec{\upsilon }}_{\mathrm{ek}}\left(z\right)=\beta \left(z\right).\vec{\tau }.e^{i\theta (z)}$


The combined geostrophic current components constitute the GlobCurrent ocean current data. This data provide 3‐hourly, global ocean currents at the surface and at 15 m depth with a 0.25° spatial resolution, covering the period 1993–2015. A comparison of the virtual Lagrangian trajectories advected using GlobCurrent to real trajectories from surface drifting buoys for the SW Indian Ocean indicates good agreement between the two (Hart‐Davis, Backeberg, Halo, Sebille, & Johannessen, 2018). Particularly, the density distribution of the real and virtual trajectories suggests that the GlobCurrent forced Lagrangian particles are appropriate for use in studies of upper ocean dynamics.

Known life history parameters of *P. homarus* (Table 1) were taken into account in simple exploratory simulations. Some 1,000 virtual particles were deployed at 12 a.m. on 1st January and on 1st June in 2009 and 2010. These two periods were selected because adult lobsters sampled in 2013 and 2014 would presumably have been drifting larvae at the time, and because January and June are the peak breeding seasons in the SW and NW Indian Oceans, respectively. Particles were restricted to the upper (<15 m deep) layer of the water column and dispersed passively over a period of 120 days (4 months). Trajectories and final locations of particles were plotted. The numbers of particles that reached the coast were quantified using custom python scripts. The ocean circulation model was based on work by Hart‐Davis et al. (2018).

**Table 1:** Known life history parameters of *P. homarus* incorporated

Geographic area	Egg‐bearing	Larval behavior	Settlement of pueruli	Reference
Laboratory (India)	Eggs developed in 22–30 days at 25–30°C	Larvae released at night or early morning Phyllosomas grew to stage V in 46–61 days Cumulative intermount period: I = 7–10 days; II = 13–20 days; IIIa = 21–25 days; IIIb = 26–30 days; IIIc = 32–38 days		Vijayakumaran, Maharajan, Rajalakshmi, Jayagopal, and Remani (2014)
NW Indian Ocean (Oman)	Protracted breeding season from May/Jun to Jan/Feb	Maximum abundance of stage I in Apr–May Larvae present in water column during productive *SE* monsoon season		Al‐Marzouqi et al. (2008) Khvorov, Piontkovski, and Popova (2012)
SW Indian Ocean (South Africa)	Egg‐bearing peaks in Nov–Feb, sharp decline in Mar–Aug Repetitive breeding (up to 4 broods/year) depending on female size Rapid embryonic development takes 29–59 days, depending on water temperature	Larval release starts in Aug and peaks in March and occurs mainly at night 4–6 month dispersal duration 9 stages defined from field samples Remain within 65 km of the shore (max = 117 km) Speculation that larvae are retained in reversing current systems inshore of the Agulhas current	Pueruli observed mainly in Apr–Jul Settle near the coast (shark nets) and on shallow inshore reefs	Berry (1971b **,** 1971a) Berry (1974b)

## 3 RESULTS

### 3.1 Genetic diversity

Summary statistics of 271 *P. homarus* individuals genotyped across 21 microsatellite loci are shown in Table 2 and in Supporting Information Tables S3A and B. Microchecker did not detect evidence for scoring errors due to stuttering or large allelic dropout, but indicated that null alleles may be present at ten loci (Orn 11, Orn 12, Orn 16, G21, G25, G27, G30, G32, G53, and G58). All loci were included in subsequent analyses because no significant difference was found between ENA‐corrected versus non‐ENA‐corrected *F*_ST_ values, overall and for each population (*p* > 0.05).

**Table 2:** *Panulirus homarus* summary statistics for microsatellite data by population. *N* = total number of individuals, *N*_A_ = mean number of alleles, *H*_O_ = observed heterozygosity, *H*_E_ = expected heterozygosity, *A*_R_ = allelic richness, PHH = *P. h. homarus*, PHM = *P. h. megasculptus*, PHR = *P. h. rubellus*

Population	*N*	*N*_A_	*H*_O_	*H*_E_	*A*_R_
All	271	5.849	0.604	0.590	5.86
OM	29	7.190	0.644	0.615	6.070
YEM	24	6.333	0.605	0.610	5.690
KEN	22	5.238	0.603	0.564	4.780
ZV	17	4.952	0.573	0.555	4.800
CH	19	5.524	0.584	0.589	5.260
XX	22	6.048	0.621	0.614	5.460
FD	29	6.619	0.644	0.626	5.660
BR	19	5.476	0.616	0.577	5.240
TM	19	5.095	0.551	0.549	4.920
SB	30	6.048	0.588	0.594	5.110
PSJ	20	5.667	0.591	0.590	5.400
MB	21	6.000	0.631	0.597	5.540
*PHH*	43	6.571	0.585	0.578	6.550
*PHM*	53	7.762	0.627	0.621	7.690
*PHR*	175	8.667	0.607	0.613	8.630

Genetic variability was high at most loci, but low at Orn 21 (PIC = 0.072) and G27 (PIC = 0.185). The N_A_ per locus ranged from 2 (locus G42) to 14 (locus Orn 12). The expected heterozygosity was higher than observed at Orn 4, Orn 11, Orn 12, Orn 16, G01, G21, G25, G27, G30, G32, G53, and G58. Following Bonferroni correction, Orn 5, Orn 12, Orn 16, Orn 17, Orn 32, G03, G21, G22, G27, G32, G36, G42, and G53 deviated significantly from HWE. However, out of the 12 populations, Orn 5 deviated significantly from HWE in 5 populations, Orn 12 in 6, Orn 16 in 3, Orn 17 in 5, Orn 32 in 1, G21 in 5, G22 in 2, G32 in 6, G36 in 2, G42 in 9, and G53 in 1 population. The analyses were also conducted after removing loci that deviated from HWE, which resulted in a smaller dataset with 8 loci from 257 individuals. The results from analyses of the smaller dataset did not contradict those obtained from using the full dataset, and hence, all loci were retained (see Supporting Information Appendix S1). At the population level, N_A_ and A_R_ were highest in Oman (*N*_A_ = 7.19, *A*_R_ = 6.07) and lowest in Zavora (*N*_A_ = 4.95, *A*_R_ = 4.8). At the subspecies level, *N*_A_ and *A*_R_ were highest in *P. h. rubellus* (*N*_A_ =8.6, *A*_R_ = 8.6).

### 3.2 Genetic structure

**Figure 2:**
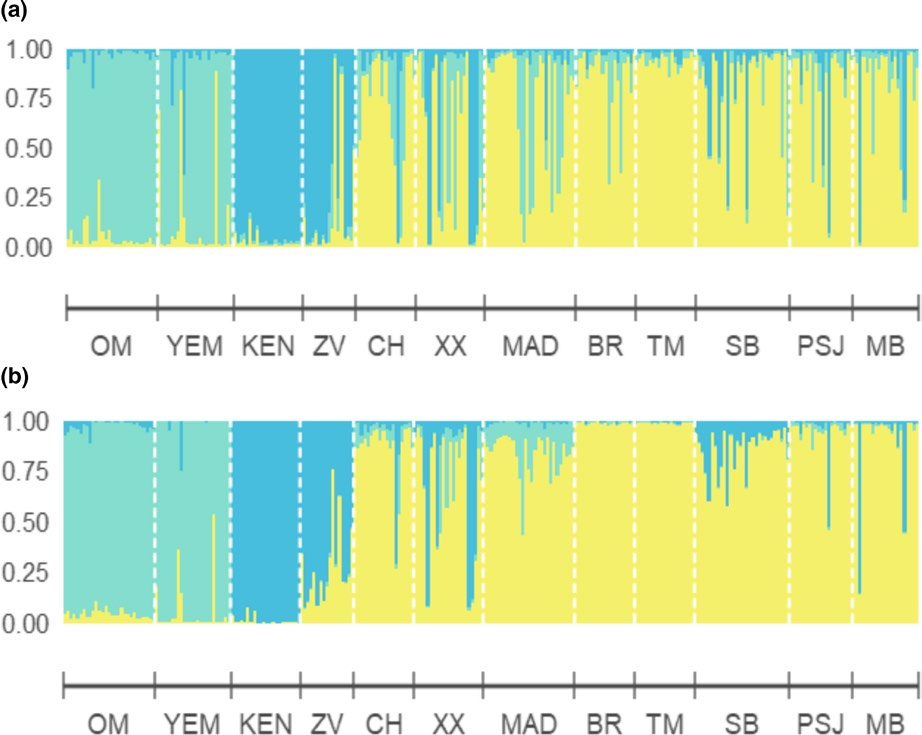
STRUCTURE plots for the *P. homarus* dataset, (a) using admixture model with no locprior and (b) admixture model with locprior

Genetic differentiation between the subspecies could clearly be distinguished using the clustering analyses implemented in STRUCTURE and Geneland. STRUCTURE analysis indicated that *K* = 3 was optimal for the *P. homarus* subspecies dataset (Figure 2a,b). The AMOVA indicated that there was 4.5% difference between each of the groups identified by the STRUCTURE analysis (Table 3). Individuals collected from the Arabian Sea (Oman and Yemen) clustered as a single population which corresponded to *P. h. megasculptus*. Similarly, individuals collected from Kenya and the northern‐most Mozambique site, Zavora, clustered together as *P. h. homarus*. The majority of specimens collected at Chidenguele and Xai Xai in Mozambique, Madagascar, and South Africa clustered as *P. h. rubellus*, although some evidence of admixture was observed.

**Table 3:** AMOVA results for the populations grouped by the STRUCTURE results. Bold values indicate statistical significance

Source of variation	Degrees of freedom	Sum of squares	Variance components	Fixation indices	% Variation
Among groups	2	100.71	0.29	**0.045**	**4.54**
Among populations within groups	13	116.11	1.48	**0.015**	**1.48**
Within populations	526	3,130.28	5.95	**0.060**	**93.98**

When comparing the genetic clusters with specimens assigned to each of the three subspecies based on their morphology (morphotypes), some evidence of admixture was observed, especially when the locprior model was not used. With locprior, all *P. h. megasculptus* morphotypes matched their corresponding genetic clusters when using locprior, but 8% showed admixture when locprior was not used. In Kenya, *P. h. homarus* morphotypes all matched the corresponding genetic clusters, irrespective of the model used. All *P. h. homarus* morphotypes collected from Zavora, Chidenguele and Xai Xai in Mozambique, and Mdumbi in South Africa also matched their respective genetic clusters, irrespective of the model used.

In Madagascar, 20% of *P. h. rubellus* morphotypes showed some evidence of admixture with *P. h. megasculptus* without locprior, but all clustered with *P. h. rubellus* when locprior was used. Of the *P. h. rubellus* morphotypes in the South African populations, 12% showed evidence of admixture with other subspecies without locprior, but when the locprior model was used, one individual from Port St Johns (5%), and another from Mdumbi (5%) corresponded to the *P. h. homarus* cluster. Out of six individuals from Zavora that were morphologically identified as *P. h. rubellus*, 4 (67%) showed a high degree of admixture with *P. h. homarus*, even when using the locprior model—thus suggesting that interbreeding and high genetic variability occurs predominantly at this location.

**Figure 3:**
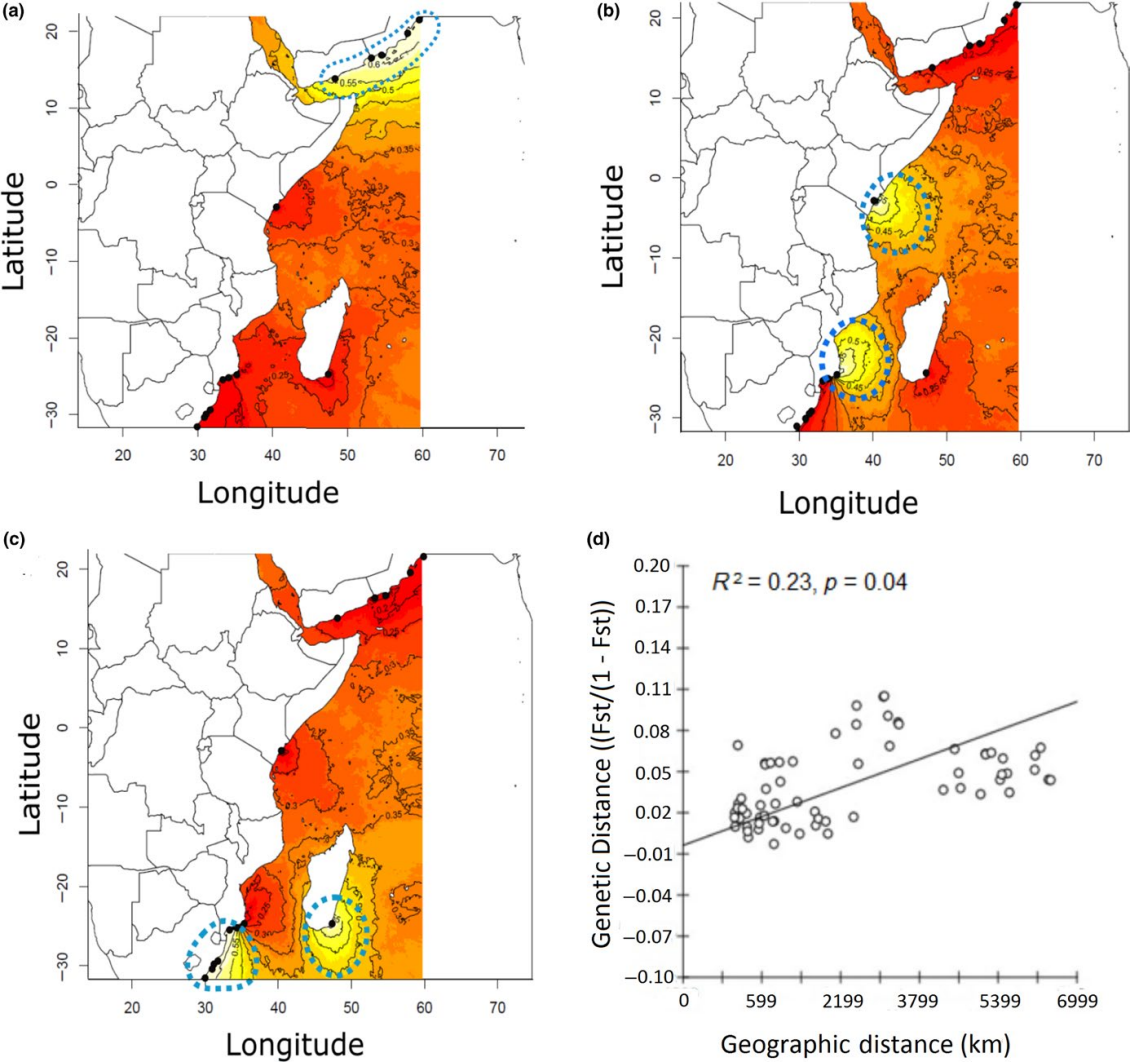
Results of the Geneland analysis. Contour plots showing the posterior probabilities of the inferred clusters corresponding to populations of (a) *P. h. megasculptus*, (b) *P. h. homarus*, and (c) *P. h. rubellus*. The highest membership values are in white to yellow, and the contour lines depict the spatial position of genetic discontinuities. (d) Isolation by distance plot showing the relationship between geographic and genetic distance

The spatially explicit Bayesian clustering model implemented in Geneland also resulted in three clear genetic clusters, corresponding to samples from Oman and Yemen (*P. h. megasculptus*; Figure 3a), Mozambique, Madagascar, and South Africa (*P. h. rubellus*; Figure 3b), and Kenya with some Mozambique samples (*P. h. homarus*; Figure 3c). Steep contour lines indicated a genetic transition zone between *P. h. homarus* and *P. h. rubellus* in southern Mozambique, where they are sympatric. Genetic and geographic distance were weakly correlated (*R* ^2^ = 0.23, *p* = 0.04) suggesting that geographic distance plays a minor role in genetic differentiation (Figure 3d).

### 3.3 Environmental factors

**Figure 4:**
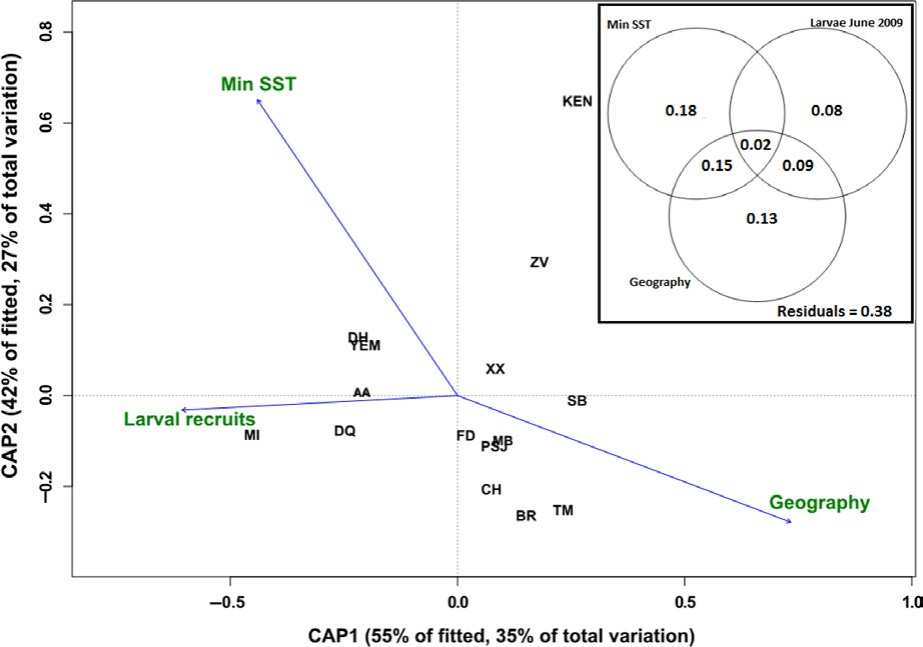
Distance‐based redundancy analysis (dbRDA) to test the effect of minimum SST, larval recruits, and geographic distance as predictor variables of genetic differentiation between the three *P. homarus* subspecies. The arrows indicate the direction of the maximum correlation, and the length of the arrows represents the strength of the correlation

The “ordistep” function identified geography, minimum SST, and larval recruits of 2009 as the most significant variables affecting the response matrix of genetic variation (Supporting Information Table S2). This model was significant when compared to the null model (*F* = 6.20, *p* = 0.001). The first axis accounted for 55% of the fitted variation and 35% of the total variation, and the second axis explained 42% of the fitted variation and 27% of the total variation (Figure 4). Variance partitioning indicated that minimum SST explained 18% of the total variation (*F* = 6.98, *p* = 0.004), and that larval recruits of June 2009 could explain 8% of the variation, but was not significant (*F* = 1.82, *p* = 0.15). Geography explained 13% of the total variation (*F* = 4.67, *p* = 0.019) and was significant (Figure 4).

### 3.4 Virtual particle dispersal simulations

#### 3.4.1 NW Indian Ocean and Kenya

**Figure 5:**
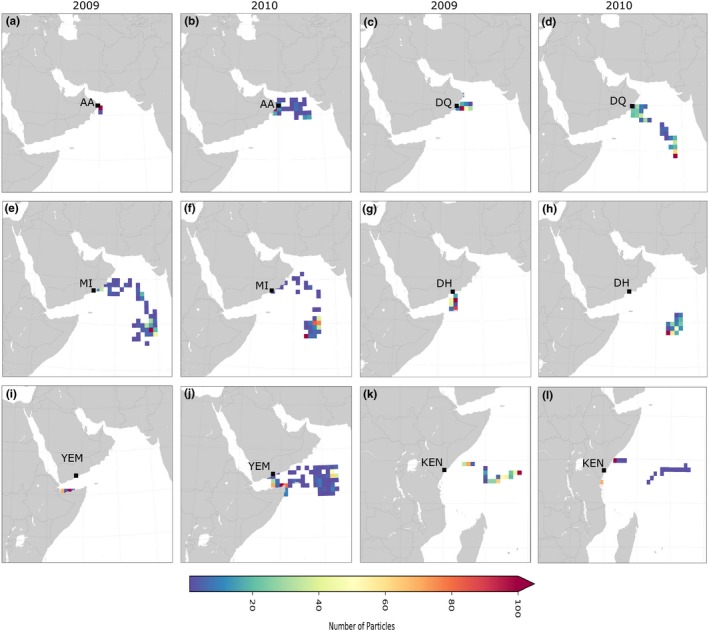
Particle density plots of the Lagrangian trajectories on a 1°× 1° spatial grid for the northern sites for the peak breeding season in June 2009 and 2010. AA: Al Ashkharah; DH: Dhalkut; DQ: Duqm; KEN: Kenya; MI: Mirbat; YEM: Yemen

At the NW Indian Ocean sites (Oman, Yemen) and Kenya, the peak breeding season of *P. homarus* is in June. Particle density plots are shown for June (Figure 5) and January (Supporting Information Figure S1) along with dispersal trajectories for January and June (Supporting Information Figures S2 and S3). Particles released in June at sites in Oman became trapped in eddies close to their release location in 2009, but dispersed wider in 2010. The proportion of released particles reaching the coast after 120 days was 30% in 2009 and 20% in 2010. Particles released in January at sites in Oman dispersed widely in offshore eddies in the Arabian Sea, and fewer of them reached the coast after 120 days, 14% in 2009 and 15% in 2010. Notably, all trajectories remained south of the Sea of Oman, where *P. homarus* adults are rarely encountered.

Particles released in Yemen in June were retained in the Gulf of Aden, with a high rate of reaching the coast (>95% in 2009 and 33% in 2010). Those released in January were advected offshore into the NW Indian Ocean, with <2% of the particles reaching the coast in either year.

Particles released in June in Kenya were retained near their release site in both years (>50% in 2010), with some dispersal northwards into Somalia in 2009. Those released in January were swept eastwards, potentially entrained in the South Equatorial Return Current. Few particles returned to the coast after 120 days (6.8% in 2009 and 9.7% in 2010), but a small proportion entered the northern Mozambique Channel in 2009. Overall, trajectories reflect dispersal within eddies and gyres, high seasonal and inter‐annual variability, and retention in eddies near release locations at several sites in June.

#### 3.4.2 SW Indian Ocean

**Figure 6:**
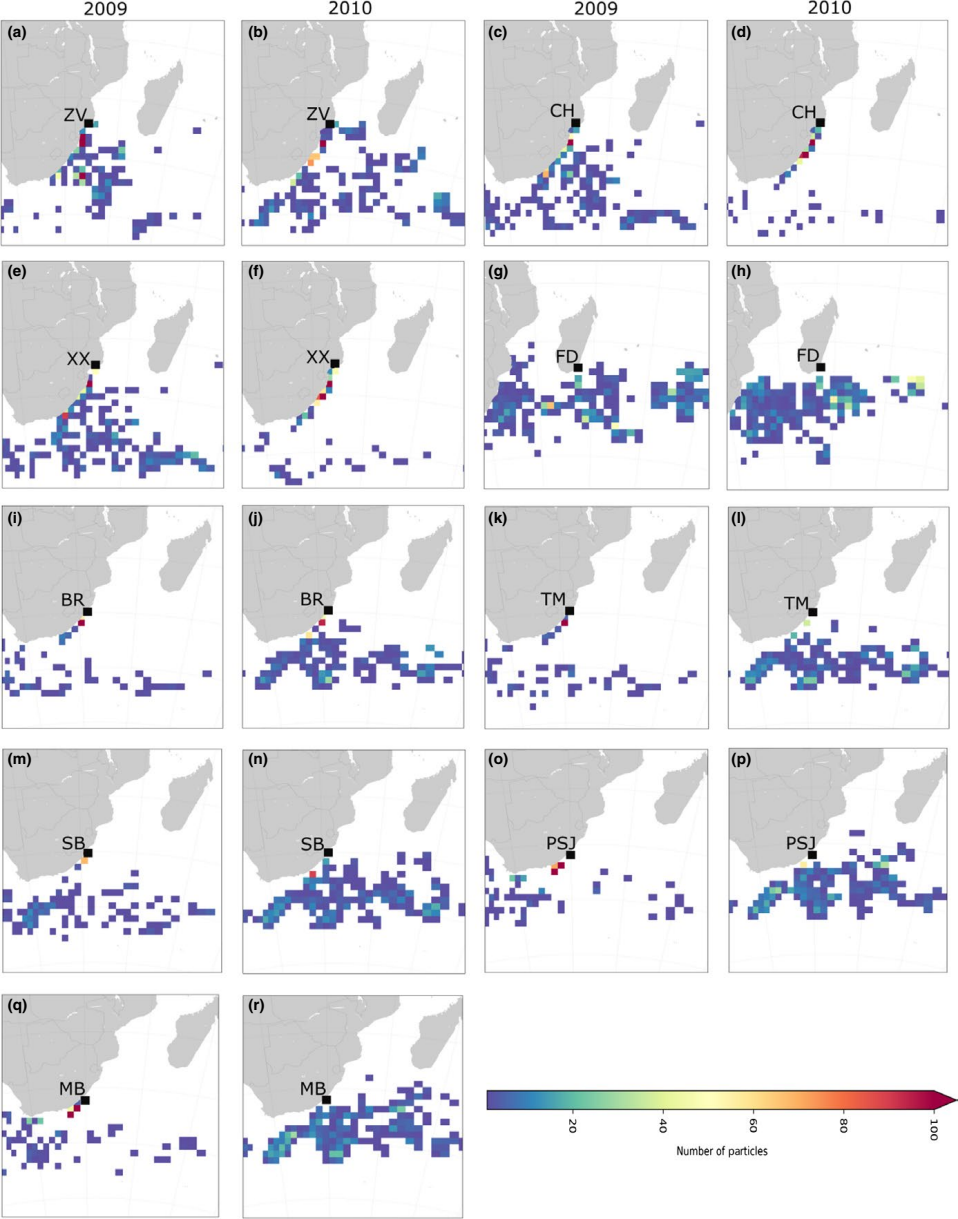
Particle density plots of the Lagrangian trajectories on a 1°× 1° spatial grid for the southern sites for the peak breeding season in January 2009 and 2010. BR: Blood Reef; CH: Chidenguele; PSJ: Port St Johns; SB: Scottburgh; MB: Mdumbi; TM: Tinley Manor; XX: Xai Xai; ZV: Zavora

Particle density plots for SW Indian Ocean sites (Mozambique, Madagascar, and South Africa) for the peak breeding season in January are shown in Figure 6. Density plots are also shown for June (Supporting Information Figure S4) along with particle trajectories for January (Supporting Information Figure S5) and June (Supporting Information Figure S6). Particles released in southern Mozambique in January were mostly swept up in the Agulhas Current, shown as southwestwards trajectories along the shelfbreak, either retroflecting eastwards into the SW Indian Ocean or leaking into the SE Atlantic basin. After 120 days, 14.9% of particles released at sites in Mozambique reached the coast in 2009 and 13% in 2010, mostly south of Zavora in Mozambique, and the majority of them in eastern South Africa. No particles reached the coast to the north of Zavora. Particles released in June showed high settlement on the coast in 2009 (62%), particularly at Chidenguele, where they were likely retained in the Delagoa Bight by a seasonal eddy. Some 12% of particles released in June 2010 reached the coast.

Particles released in southeast Madagascar in both years and seasons tracked across the Mozambique Channel toward the mainland coast of southern Mozambique and eastern South Africa, and most became entrained in eddies, remaining in the channel or south of it after 120 days. Although some particles released in January reached the mainland coast after 120 days (1.6% in 2009 and 1.7% in 2010), many were close to it, just off the shelf. Some 2% of particles released in January were returned to southern Madagascar, and of those released in June, 1.2% were returned.

Particles released in January at all sites in eastern South Africa were advected southwestwards by the Agulhas Current, and either reached the coast, retroflected into the SW Indian Ocean, or leaked into the SE Atlantic after 120 days. Some 13.4% of particles released at Blood Reef reached the coast after 120 days in 2009 and 10.2% in 2010, mostly to the south of their release locations. Particles released from the southernmost sites rarely reached the coast after 120 days, suggesting that populations occurring here receive larvae from sites further to the north. Particles released in June (outside the main breeding season) were dispersed further offshore than those released in January at nearly all sites. On average, particles released in January at South African sites reached the coast in 10% (2009) and 5% (2010) of cases after 120 days, compared to <1% (2009) and 6% (2010) of particles released in June. High seasonal and inter‐annual variability in dispersal patterns were also observed in the SW Indian Ocean region, with evidence that particles can become trapped and retained in eddies near, or downstream of their release locations, despite the narrow shelf and proximity of the Agulhas Current along the shelf‐edge.

## 4 DISCUSSION

### 4.1 Genetic structure and boundary zones

We examined the fine‐scale population genetic structure and phylogeography of *P. homarus* in the Western Indian Ocean, based on fast‐evolving nuclear microsatellite markers. We also used a seascape genetics approach to relate the observed genetic structure to oceanographic influences. Three clear genetic clusters corresponded to samples from the Arabian Sea (NW Indian Ocean; *P. h. megasculptus),* SW Indian Ocean (*P. h. rubellus*), and the more tropical region in‐between (*P. h. homarus*). A contact zone between *P. h. homarus* and *P. h. rubellus* near Zavora (in the Delagoa Bight, Mozambique) was inferred from the high degree of admixed individuals originating from Zavora in the STRUCTURE analysis, and from the genetic transition zone in the Geneland analysis.

The microsatellite analysis suggests that *P. h. homarus* (Kenya) and *P. h. megasculptus* (Oman and Yemen) are evolutionarily distinct (*F*_ST_ > 0.07, Supporting Information Table S4). Farhadi et al. (2013, 2017 ) also found evidence of genetic divergence between Arabian Sea and more equatorial *P. homarus* populations, based on hypervariable control region sequences and microsatellites. The two groups are morphologically distinguishable (microsculpta vs. macrosculpta forms) and inhabit distinct geographic ranges, with some overlap in Oman (J. Groeneveld, personal communication). Nevertheless, more conserved mitochondrial and nuclear DNA markers could not differentiate the two groups (Lavery et al., 2014; Singh et al., 2017), thus suggesting a recent divergence.

The simulated particle trajectories strongly support the retention of *P. h. megasculptus* larvae within the NW Indian Ocean. Final locations after 120 days suggest that a proportion of larvae will reach, and presumably recruit back to populations in the Gulf of Aden and in the Arabian Sea. Mesoscale eddies in the Gulf of Aden are likely to retain larvae released in June within the Gulf, but the eddies weaken seasonally (Bower & Furey, 2012), giving rise to eastward dispersal of particles released in January, into the Arabian Sea. *Panulirus homarus megasculptus* in Oman has a prolonged breeding season with multiple broods (Al‐Marzouqi, Al‐Nahdi, Jayabalan, & Groeneveld, 2007; Al‐Marzouqi, Groeneveld, Al‐Nahdi, & Al‐Hosni, 2008), and drifting larvae are expected to be present in the Arabian Sea throughout the year. Ocean circulation is influenced by the seasonally reversing Somali Current and eddies which develop along the coasts of Oman, Yemen, and Somalia (Schott & McCreary, 2001). These eddies likely retain larvae in the NW Indian Ocean, thereby facilitating the incipient genetic structure observed between *P. h. megasculptus* and *P. h. homarus* (our results and see also Farhadi et al., 2013). The oceanographic boundary is not restricted to dispersal of lobster larvae and also prevents northwards dispersal of starfish *Acanthaster planci* larvae from east Africa into the northern Arabian Sea (Vogler et al., 2012).

For the SW Indian Ocean populations, spatially explicit clustering provided empirical support for a barrier to gene flow between *P. h. homarus* and *P. h. rubellus* near the Delagoa Bight, where they are sympatric. Isolation by distance did not play a crucial role in influencing population genetic structure among the subspecies. For example, *P. h. homarus* at Zavora (northern edge of Delagoa Bight) were more closely related to those in Kenya, thousands of kilometers away (*F*_ST_ = 0.016, Supporting Information Table S4), and Xai Xai which is 169 km to the south (*F*_ST_ = 0.015), than to those in Chidenguele (122 km south of Zavora, *F*_ST_ = 0.066). This provides strong evidence for the presence of a phylogeographic barrier between subspecies in the Delagoa Bight.

### 4.2 Environmental drivers of genetic discontinuities

The multivariate analysis dbRDA was used to investigate the correlation between genetic variation, environmental, and spatial factors across the seascape. Minimum SST was correlated with genetic variability (Figure 4), suggesting that ambient temperature may affect the success of larval dispersal. Coastal waters become progressively cooler toward the south (i.e., southern Mozambique and eastern South Africa), potentially playing a selective role by reducing the fitness of *P. h. homarus* larvae originating from more tropical waters to the north of Zavora in Mozambique. Nevertheless, low densities of *P. h. homarus* do occur in eastern South Africa—potentially originating from Zavora or further north, and they must have survived the temperature gradient associated with southwards dispersal into cooler coastal waters. The absence of definitive evidence for hybrids between the two forms supports a hypothesis of specific mating and spawning preferences that may be spatially and/or temporally distinct, and potentially maintained through adaptation and behavioral reproductive isolation (Farhadi et al., 2017). Van der Meeren, Chandrapavan, and Breithaupt (2008) demonstrated similar behavioral mechanisms among clawed lobsters *Homarus gammarus* and introduced *H. americanus* in the NE Atlantic.

### 4.3 A biogeographic perspective

From a biogeographic perspective, *P. h. homarus* and *P. h. megasculptus* inhabit the Western Indo‐Pacific region, whereas *P. h. rubellus* is restricted to the southwestern edge of the region, spilling over into the temperate Southern African region. At a finer resolution, *P. h. rubellus* extends southwards from the Delagoa Bight ecoregion in Mozambique, to the Natal and Agulhas Bank ecoregions in South Africa, and the Southeast Madagascar ecoregion (Spalding et al., 2007). We propose the Delagoa Bight ecoregion as a contact zone between *P. h. homarus* and *P. h. rubellus*. The Bight is a coastal indentation at the southern end of the Mozambique Channel, and both its location and unique oceanographic features may have contributed to the genetic discontinuity seen here. The Bight is affected by eddies moving gradually southwards through the Mozambique Channel, and also by cross‐channel eddies originating from the East Madagascar Current, on its path around the southern tip of Madagascar (Cossa, Pous, Penven, Capet, & Reason, 2016; Lutjeharms, 2006). Additionally, the Bight is located near the upper reaches of the Agulhas Current (Lutjeharms, 2006), and particles released here were frequently advected southwestwards.

A quasi‐stationary and topographically induced Delagoa Bight Eddy (Cossa et al., 2016; Halo et al., 2017; Lamont, Roberts, Barlow, Morris, & Berg, 2010; Lutjeharms & da Silva, 1988; Saetre & da Silva, 1984) creates strong upwelling and a cool Delagoa Bight cell, with near‐surface temperatures of <16°C at 150 m depth. The cell can potentially form a boundary that restricts gene flow between the two subspecies, but it is intermittent and therefore unlikely to be impermeable. A mixture of equatorial and subtropical waters in this area may also contribute to the contact zone, facilitating settlement of postlarvae of *P. h. homarus* originating from further north, and of *P. h. rubellus* originating in the Bight or from southeast Madagascar.

Teske et al. (2009) identified a tropical/subtropical phylogeographic break near Cape St Lucia (some 200 km south of the Delagoa Bight) based on data from estuarine prawns. These authors proposed a selection gradient resulting from the diminishing effects of the Agulhas Current on nearshore waters to the south of St Lucia, where the Natal Bight deflects the current away from the coast, resulting in lower SSTs and potential changes in circulation patterns, such as the formation of eddies and nearshore return currents (Teske et al., 2011). Our study suggests a similar genetic break, acting on spiny lobster larvae, but further to the north, at the Delagoa Bight.

### 4.4 Influence of ocean currents on gene flow and connectivity

A large, seasonal anticyclonic cell prevails at the northern entrance to the Mozambique Channel (Donguy & Piton, 1991), followed by a succession of mesoscale cyclonic and anticyclonic eddies propagating southwards through the channel (Collins, Hermes, & Reason, 2014; Collins, Hermes, Roman, & Reason, 2016; Halo et al., 2017). These eddies create strong dynamic gradients which act on larval dispersal. Silva, Mesquita, and Paula (2010) speculated that the anticyclonic circulation pattern in the channel randomizes larval dispersal in the mangrove crab *Perisesarma guttatum*, resulting in panmictic populations to the north of the channel. Similarly, Madeira, Judite Alves, Mesquita, Silva, and Paula (2012) suggested that the upper Agulhas Current transports *Cerithidea decolata* larvae southwards, thus homogenizing populations in southeast Africa.

The genetic structure in *P. homarus* in the SW Indian Ocean is similarly attributed to the prevailing current systems in the Mozambique Channel. Evidence that populations to the north of the channel are differentiated from those at the south has been documented for shallow‐water prawns *Fenneropenaeus indicus* and *Metapenaeus monoceros* (Mkare, Groeneveld, Teske, & Matthee, 2017), green turtle *Chelonia mydas* (Bourjea et al., 2007), blotcheye soldierfish *Myripristis berndti* (Muths et al., 2011), honeycomb grouper *Epinephelus merra* (Muths, Tessier, & Bourjea, 2015), and coral species *Acropora austera* and *Platygyra daedalea* (Montoya‐Maya, Schleyer, & Macdonald, 2016).

Particles released at Mozambican sites in the peak January breeding season of *P. h. rubellus* were often entrained in the Agulhas Current or its inshore filaments, and dispersed southwestwards. Some particles retroflected eastwards into the SW Indian Ocean (presumably lost) or leaked into the SE Atlantic basin through ring shedding, eddies, and filaments (Gordon, 2003). A proportion of particles released at Mozambican sites reached the coast, mostly near their release locations in southern Mozambique or in eastern South Africa, suggesting that many *P. h. rubellus* postlarvae that settle off eastern South Africa may originate from Mozambique. Overall, the high seasonal and inter‐annual variability in dispersal patterns is expected to result in genetic panmixia of *P. h. rubellus* (see Madeira et al., 2012). From a fisheries management perspective, genetic panmixia implies *that P. h. rubellus* in eastern South Africa and southern Mozambique may be considered a shared resource.

Particles released in southeast Madagascar could not fully explain the connectivity between Madagascar and African shelf populations, potentially because particles are assumed to be passive drifters in the Lagrangian model. In reality, spiny lobster phyllosomas are able to swim and position themselves in the water column to benefit from water movements at different depths (Bradford et al., 2005; Chiswell & Booth, 1999, 2005, 2008). Vertical migrations to deeper habitats during the day would reduce predation, with phyllosomas rising to the surface at night to feed (Bradford et al., 2005; Butler et al., 2011). Directional swimming is also not included in the particle model. Jeffs, Montgomery, and Tindle (2005) demonstrated that late‐stage larvae receive sensory cues directing them toward the shore; cues may include acoustic reef sounds (Hinojosa et al., 2016), chemical (Hadfield & Paul, [Link]), celestial, magnetic field, or electrosense signals (Jeffs et al., 2005). The postlarval puerulus stage is also a strong swimmer, able to cross the shelf using stored energy reserves (Phillips & Olsen, 1975; Wilkin & Jeffs, 2011). It is therefore clear that behavior will affect the dispersal of larvae (Butler et al., 2011). Particles released in southeast Madagascar were propagated across the Mozambique Channel on several occasions, reaching the African shelf. Directed swimming by larvae dispersed to here may have assisted them in reaching coastal settlement habitats in Mozambique or eastern South Africa.

Migrations and spawning behavior of adult spiny lobsters may mitigate the influence of the Agulhas Current on the downstream dispersal of larvae (Groeneveld & Branch, 2002; Santos, Rouillard, & Groeneveld, 2014), through determining the location and timing of larval release. Vertical migration of larvae in the water column has been shown to favor larval retention close to local shores (Phelps, Polton, Souza, & Robinson, 2015; Rivera et al., 2013). Teske, Sandoval‐Castillo, Sebille, Waters, and Beheregaray (2015), Teske, Sandoval‐Castillo, Sebille, Waters, and Beheregaray (2016) showed that larvae can remain close to the shore and be dispersed by inshore counter‐currents. We suggest that *P. homarus* larvae can position themselves to avoid being caught up in the main Agulhas Current, and are instead dispersed by inshore counter‐currents. This observation is supported by a considerable proportion of particles reaching the coast near, or a moderate distance to the southwest of their release locations, after 120 days at large. Had they all been caught up in the Agulhas Current, we suggest that none, or very few of them would have been returned to the coast.

The particle model used here may overstate the magnitude of larval dispersal. The spatial resolution of the GlobCurrent model is 25 km, and coastal submesoscale processes are therefore not resolved. Fine‐scale hydrographic processes that may “push” or “pull” particles toward or away from the coast are most likely crucial in determining larval settlement patterns, but could not be resolved with the model used. While global operational ocean models (e.g., https://hycom.org/or https://marine.copernicus.eu/) are approaching spatio‐temporal resolutions to resolve submesoscale processes, they are unable to represent the correct levels of variability, particularly in the highly dynamic Agulhas Current (Meyer, Braby, Krug, & Backeberg, 2017).

Particle tracking simulations were constrained to January and June to provide seasonal contrast to dispersal patterns. The simulations included the peak breeding seasons of *P. h. rubellus* in the SW Indian Ocean (January) and of *P. h. megasculptus* and *P. h. homarus* in the NW Indian Ocean and Kenya (June). Simulations were further restricted to 2009 and 2010 and showed considerable inter‐annual variability—a factor that has been implicated in recruitment patterns of species with drifting larvae (Botsford, 2001; Chelton, Bernal, & McGowan, 1982; Griffin, Wilkin, Chubb, Pearce, & Caputi, 2001). *Panulirus homarus* females can carry up to four batches of eggs per year, releasing them during different months (Al‐Marzouqi et al., 2008), when oceanographic conditions would differ from those in the models. The present particle model can therefore only provide a broad indication of larval dispersal patterns. Nevertheless, the close correlation between its outputs and the observed genetic structure provides new insights into the scale of larval dispersal and settlement of *P. homarus* in the Western Indian Ocean.

In conclusion, a seascape genetics approach illustrated that complex physical oceanography and biogeographic boundaries could explain genetic diversity among *P. homarus* subspecies, along with isolation by distance. Eddies in the Arabian Sea potentially retain larvae in that region, thus contributing to the differentiation between *P. h. megasculptus* and *P. h. homarus*. A steep genetic gradient between *P. h. homarus* and *P. h. rubellus* was demonstrated in the Delagoa Bight ecoregion in Mozambique. Virtual particles released south of the Bight were advected southwestwards by boundary currents, or reached the coast near or downstream of their release locations. Minimum SST was correlated with genetic variability, suggesting ambient temperature and larval tolerance as selective factors in dispersal success. Ocean currents and SST may play key roles in maintaining the cooler subtropical and warmer tropical distribution ranges of *P. h. rubellus* and *P. h. homarus* in the SW Indian Ocean.

## CONFLICT OF INTEREST

None declared.

## AUTHOR CONTRIBUTIONS

S.P.S., J.C.G., and S.W.‐M. conceived the sampling design; S.P.S. conducted laboratory work; S.P.S. and M.G.H.‐D. conducted analyses and wrote the manuscript; J.C.G., S.W.‐M., and B.C.B. contributed to versions of the manuscript.

## DATA ACCESSIBILITY

Data are available from the Dryad Digital Repository: https://doi.org/10.5061/dryad.hf068m7.
