# Supplementary material for: Seascape genetics of the spiny lobster Panulirus homarus in the Western Indian Ocean: Understanding how oceanographic features shape the genetic structure of species with high larval dispersal potential
Source: Ecol Evol. 2018 Nov 16;8(23):12221–37. doi: 10.1002/ece3.4684 (PMC6303728; doi:10.1002/ece3.4684)
Supplement: Supplementary file 1 [file ECE3-8-12221-s001.docx]

**Supplementary analyses**

**Re-analysis of data using 8 loci (loci that did not deviate from Hardy-Weinberg Equilibrium in any population)**

Individuals with more than 20% data missing (data missing at more than 1 locus) were removed, resulting in a dataset containing 257 individuals.

1. Pairwise F_ST_ values. Values in bold indicate statistical significance (*p* < 0.05)

|  | 1 | 2 | 3 | 4 | 5 | 6 | 7 | 8 | 9 | 10 | 11 | 12 |
| --- | --- | --- | --- | --- | --- | --- | --- | --- | --- | --- | --- | --- |
| 1. OM | 0 |  |  |  |  |  |  |  |  |  |  |  |
| 2. YEM | 0 | 0 |  |  |  |  |  |  |  |  |  |  |
| 3. KEN | **0.067** | **0.083** | 0 |  |  |  |  |  |  |  |  |  |
| 4. ZV | **0.073** | **0.088** | 0.011 | 0 |  |  |  |  |  |  |  |  |
| 5. CH | **0.070** | **0.091** | **0.095** | **0.102** | 0 |  |  |  |  |  |  |  |
| 6. XX | **0.039** | **0.044** | **0.036** | **0.028** | **0.023** | 0 |  |  |  |  |  |  |
| 7. FD | **0.032** | **0.038** | **0.070** | **0.077** | **0.023** | **0.008** | 0 |  |  |  |  |  |
| 8. BR | **0.060** | **0.085** | **0.088** | **0.077** | 0.011 | **0.026** | **0.022** | 0 |  |  |  |  |
| 9. TM | **0.058** | **0.055** | **0.062** | **0.044** | **0.021** | -0.004 | 0.014 | 0.011 | 0 |  |  |  |
| 10. SB | **0.047** | **0.053** | **0.057** | **0.053** | **0.016** | -0.002 | 0.002 | **0.017** | -0.005 | 0 |  |  |
| 11. PSJ | 0.008 | **0.030** | **0.047** | **0.052** | **0.019** | 0.012 | 0.005 | 0.007 | 0.008 | 0.004 | 0 |  |
| 12. MB | **0.044** | **0.044** | **0.060** | **0.058** | **0.020** | -0.005 | 0.006 | 0.014 | -0.016 | -0.001 | 0.005 | 0 |

There was high genetic differentiation between *P. h. homarus* in Kenya and Zavora and *P. h. megasculptus* in Oman and Yemen (F_ST_ > 0.06). *P. h. rubellus* populations were also differentiated from *P. h. homarus* populations (F_ST_ > 0.05 in Chidenguele, Fort Dauphin, Blood Reef, Tinley Manor, Scottburgh and Mdumbi) and from the *P. h. megasculptus* group (F_ST_ > 0.05 in Chidenguele, Blood Reef, Tinley Manor, Scottburgh).


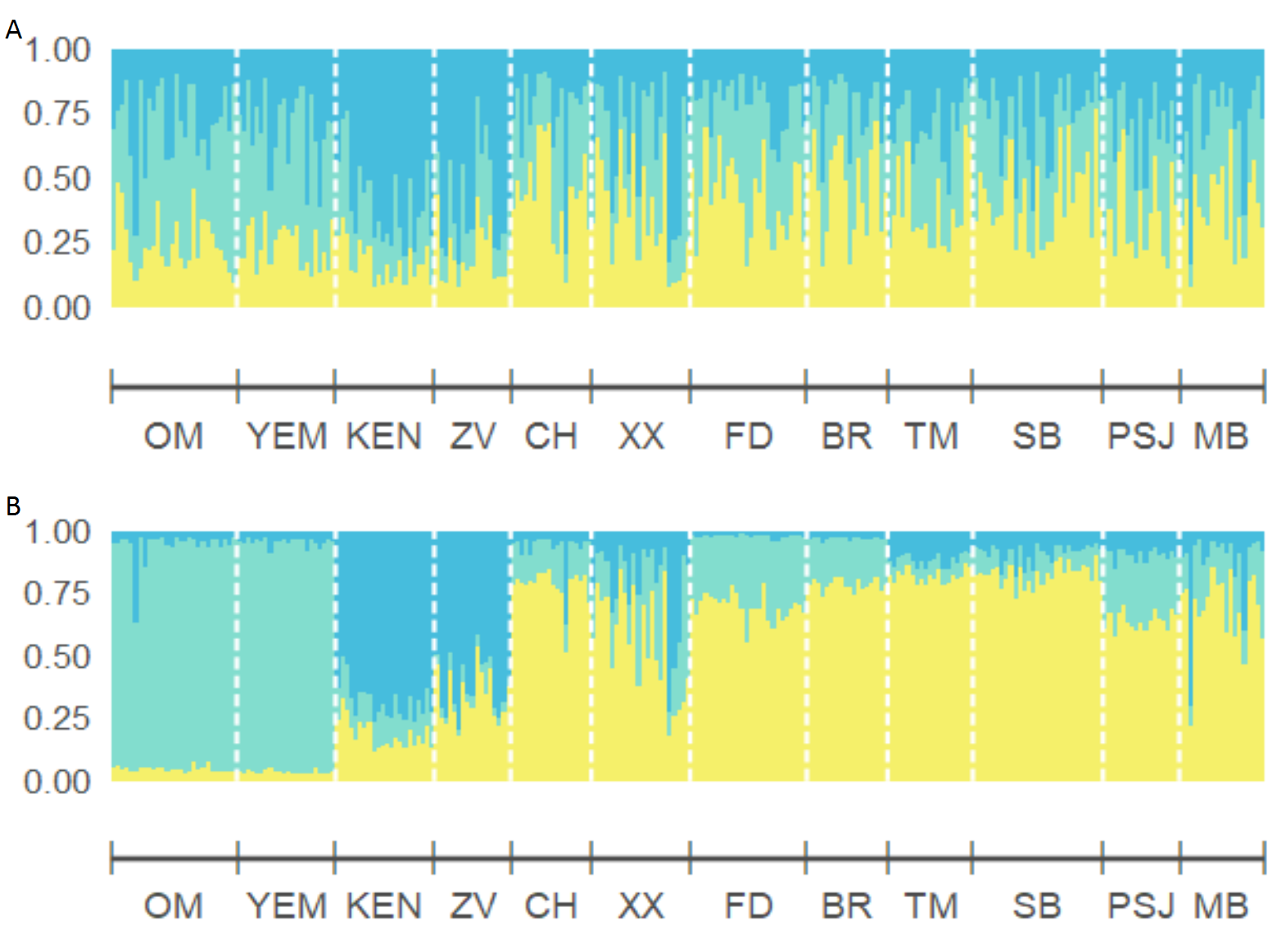


Figure 1. STRUCTURE plot using the 8-loci datset (A) without the locprior model and (B) the locprior model. The best number of clusters detected by the Evanno method was K = 3.

A high degree of admixture was observed when using a model with no locprior. When the locprior model was used, the genetic clusters of the three subspecies were more clearly distinguished.

Table 2. AMOVA results for the populations grouped by the STRUCTURE results. Bold values indicate statistical significance (*p* < 0.05).

| Source of Variation | Degrees of Freedom | Sum of Squares | Variance components | Fixation indices | Percentage of variation |
| --- | --- | --- | --- | --- | --- |
| Among groups | 2 | 38.57 | 0.11 | **0.048** | **4.76** |
| Among populations within groups | 13 | 39.44 | 0.02 | **0.011** | **1.03** |
| Within populations | 526 | 1183.10 | 2.25 | **0.058** | **94.21** |

The AMOVA results indicate that the variation among the groups partitioned in the STRUCTURE analysis was 4.76% and statistically significant.


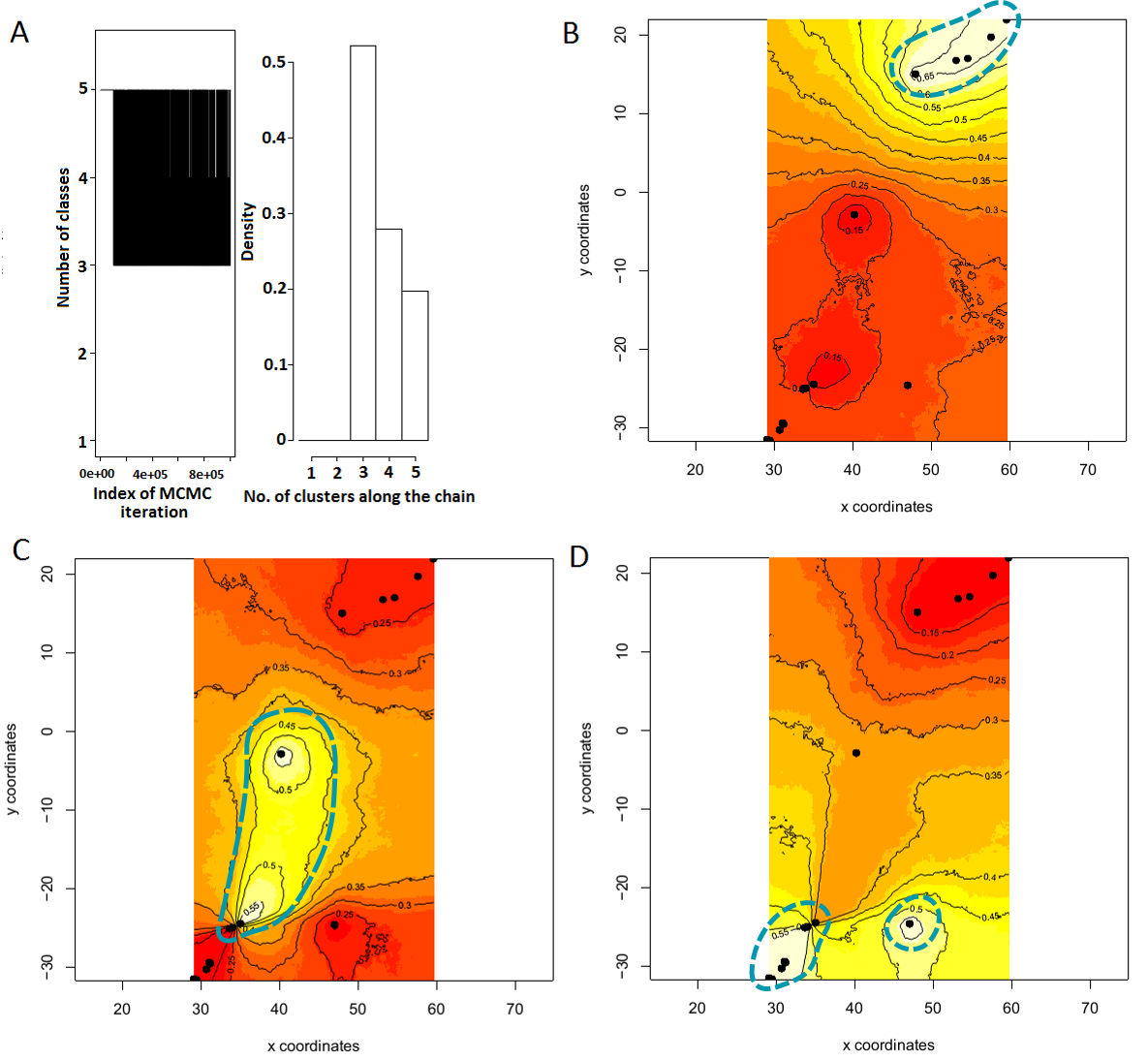


Figure 2. Results of the Geneland analysis. (A) Posterior distribution of the number of genetic clusters, showing a clear mode at K = 3. (B) Contour plot showing the posterior probabilities of the inferred clusters corresponding to *P. h. megasculptus* populations, (C) *P. h. homarus*, (D) *P. h. rubellus*. The highest membership values are in white to yellow, and the contour lines depict the spatial position of genetic discontinuities.

The spatially explicit Bayesian clustering model implemented in Geneland also resulted in three clear genetic clusters (Figure 2A), corresponding to samples from Oman and Yemen (*P. h. megasculptus*; Figure 2B), Kenya with some Mozambique samples (*P. h. homarus*; Figure 2C) and Mozambique, Madagascar and South Africa (*P. h. rubellus*; Figure 2D) . Steep contour lines indicated a genetic transition zone between *P. h. homarus* and *P. h. rubellus* in southern Mozambique, where they are sympatric*.*


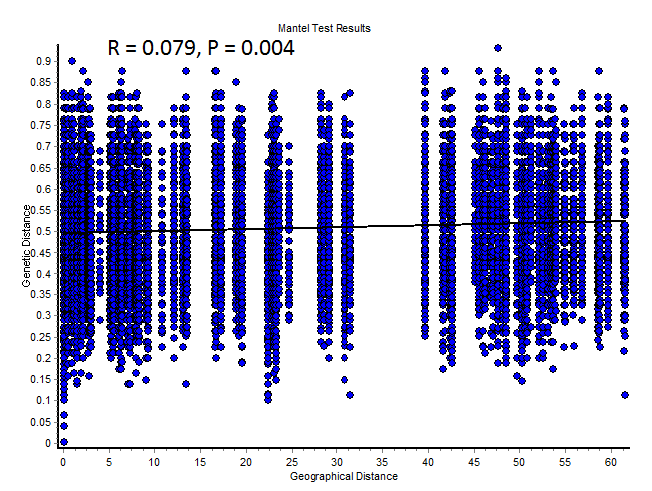


Figure 3. Isolation by Distance plot generated using the program Alleles in Space. There is a weak correlation between genetic and geographic distance.

Genetic and geographical distance were weakly correlated (R = 0.079, p = 0.04) suggesting that geographic distance plays a minor role in genetic differentiation.

Table 3. Significance tests for predictor variables used in the distance-based redundancy analysis.

| Predictor | DF | AIC | F | P-value |
| --- | --- | --- | --- | --- |
| **Geography** | **1** | **-58.232** | **6.3915** | **0.005** |
| **Min SST** | **1** | **-55.837** | **3.5298** | **0.02** |
| **Larval recruits June 2009** | **1** | **-55.503** | **3.1665** | **0.03** |
| KD420 (Turbidity) | 1 | -53.315 | 0.9718 | 0.395 |
| Larval recruits June 2010 | 1 | -53.04 | 0.7182 | 0.57 |
| Larval recruits January 2010 | 1 | -53.059 | 0.7357 | 0.58 |
| Larval recruits January 2009 | 1 | -52.845 | 0.5412 | 0.655 |

Geography, min SST and the larval recruits of the 2009 particle dispersal simulation were identified as significant predictors and were therefore used in the distance-based redundancy analysis.


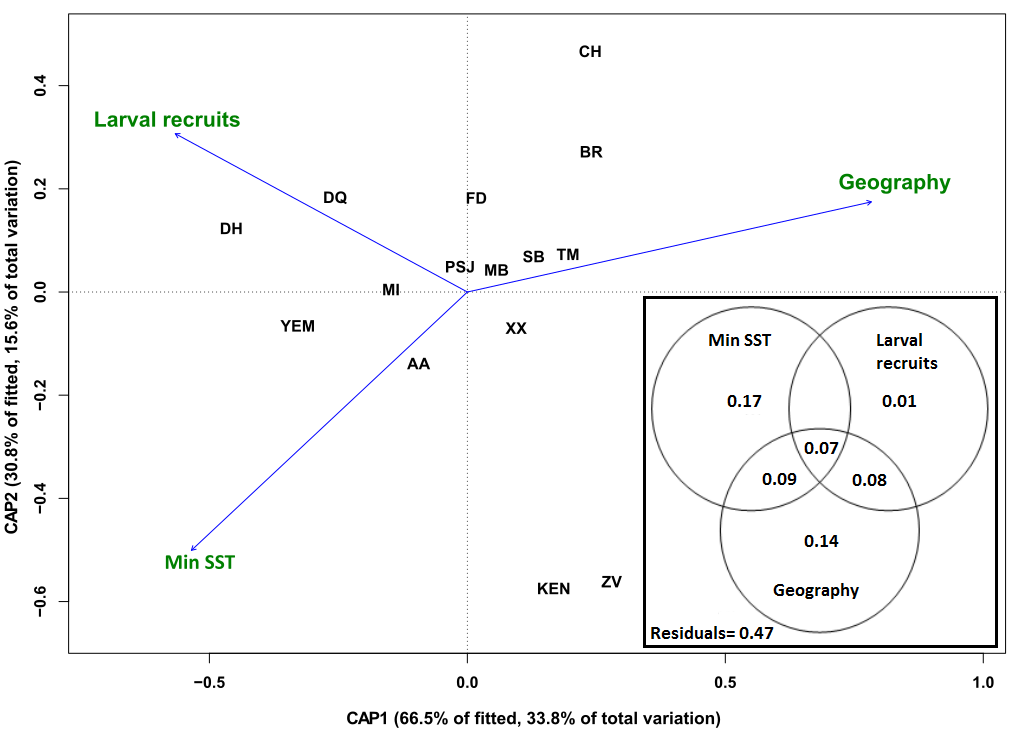


Figure 5. Distance-based redundancy analysis (dbRDA) to test the effect of minimum SST, larval recruits and geographic distance as predictor variables of genetic differentiation between the three *P. homarus* subspecies. The arrows indicates the direction of the maximum correlation and the length of the arrows represents the strength of the correlation.

The full model with min SST, larval recruits of June 2009 and geography was significant when compared to the null model (F = 3.78, p = 0.002). The first axis accounted for 66.5% of the fitted variation and 33.8% of the total variation, and the second axis explained 30.8% of the fitted variation and 15.6 of the total variation. Variance partitioning indicated that min SST explained 17% of the total variation (F = 2.21, p = 0.085), the larval recruits of June 2009 simulation could explain only 1% of the total variation (F = 1.79, p = 0.138) but was not significant. Geography explained 14% of the total variation (F = 2.95, p = 0.045).
