# Supplementary material for: Seascape genetics of the spiny lobster Panulirus homarus in the Western Indian Ocean: Understanding how oceanographic features shape the genetic structure of species with high larval dispersal potential
Source: Ecol Evol. 2018 Nov 16;8(23):12221–37. doi: 10.1002/ece3.4684 (PMC6303728; doi:10.1002/ece3.4684)
Supplement: Supplementary file 2 [file ECE3-8-12221-s002.docx]

**Supplementary tables & figures**

Table S1. Multiplex PCR primer combinations for the microsatellites.

| **Multiplex** | **Primers** |
| --- | --- |
| A | Orn 4, Orn 16, Orn 21 |
| B | Orn 5, Orn 11 |
| C | Orn 12 and Orn 32 |
| D | Orn 17 |
| E | G01, G32, G35, G36, G53 |
| F | G03, G21, G42, G58 |
| G | G22, G25, G27, G30 |

Table S2. Selection of the most informative variables used in the distance-based redundancy analysis.

| **Predictor variable** | **Df** | **AIC** | **F** | **Pr(>F)** |
| --- | --- | --- | --- | --- |
| Geography | 1 | -57.706 | 6.4595 | 0.005 |
| Min SST | 1 | -56.75 | 5.2588 | 0.005 |
| LarvaeJun09 | 1 | -55.19 | 3.4549 | 0.010 |
| LarvaeJan10 | 1 | -53.39 | 1.5937 | 0.19 |
| LarvaeJun10 | 1 | -52.812 | 1.0425 | 0.34 |
| LarvaeJan09 | 1 | -52.78 | 1.0125 | 0.345 |
| KD420 (Turbidity) | 1 | -52.519 | 0.7711 | 0.38 |

Table S3 A. Microsatellite summary statistics by population and locus for the Dao et al. (2013) primer set. N = number of individuals, Na = number of alleles, Ar = allelic richness, Ho = observed heterozygosity, He = expected heterozygosity, p(HWE) = probability of deviation from HWE, PHH = *P. h. homarus*, PHM = *P. h. megasculptus*, PHR = *P. h. rubellus*, PIC = polymorphic information content.

| **Location/ Subspecies** | **N** |  | **Orn 4** | **Orn 5** | **Orn 11** | **Orn 12** | **Orn 16** | **Orn 17** | **Orn 21** | **Orn 32** |
| --- | --- | --- | --- | --- | --- | --- | --- | --- | --- | --- |
| **All** | 271 | **Na** | 4.167 | 7.750 | 11.167 | 14.00 | 7.583 | 4.000 | 1.917 | 2.250 |
|  |  | **Ar** | 4.087 | 7.685 | 10.548 | 13.62 | 7.338 | 4.600 | 1.964 | 3.015 |
|  |  | **Ho** | 0.651 | 0.848 | 0.812 | 0.768 | 0.554 | 0.934 | 0.079 | 0.408 |
|  |  | **He** | 0.660 | 0.833 | 0.884 | 0.925 | 0.761 | 0.619 | 0.077 | 0.348 |
|  |  | **p(HWE)** | 0.962 | **0** | 0.058 | **0** | **0** | **0** | 1 | **0** |
|  |  |  |  |  |  |  |  |  |  |  |
| **OM** | 29 | **Na** | 5 | 7 | 13 | 18 | 7 | 6 | 2 | 3 |
|  |  | **Ar** | 4.403 | 6.324 | 10.98 | 14.06 | 6.13 | 4.86 | 1.517 | 2.513 |
|  |  | **Ho** | 0.538 | 0.964 | 0.857 | 0.483 | 0.517 | 0.964 | 0.034 | 0.276 |
|  |  | **He** | 0.683 | 0.758 | 0.908 | 0.926 | 0.739 | 0.677 | 0.034 | 0.246 |
|  |  | **p(HWE)** | 0.132 | **0** | **0** | **0** | 0.015 | **0** | 1 | 1 |
|  |  |  |  |  |  |  |  |  |  |  |
| **YEM** | 24 | **Na** | 4 | 8 | 9 | 14 | 7 | 5 | 2 | 4 |
|  |  | **Ar** | 3.983 | 7.309 | 8.433 | 12.04 | 5.859 | 4.533 | 1.75 | 3.304 |
|  |  | **Ho** | 0.917 | 0.909 | 0.773 | 0.565 | 0.542 | 0.957 | 0.05 | 0.652 |
|  |  | **He** | 0.677 | 0.838 | 0.852 | 0.913 | 0.743 | 0.650 | 0.05 | 0.492 |
|  |  | **p(HWE)** | 0.032 | **0** | 0.083 | **0** | 0.057 | **0** | 1 | 0.125 |
|  |  |  |  |  |  |  |  |  |  |  |
| **KEN** | 22 | **Na** | 4 | 4 | 13 | 13 | 7 | 6 | 2 | 3 |
|  |  | **Ar** | 3.904 | 3.429 | 10.64 | 11.81 | 5.949 | 5.99 | 1.998 | 3 |
|  |  | **Ho** | 0.714 | 0.524 | 0.864 | 1 | 0.409 | 1 | 0.227 | 0.909 |
|  |  | **He** | 0.617 | 0.502 | 0.864 | 0.909 | 0.751 | 0.818 | 0.206 | 0.609 |
|  |  | **p(HWE)** | 0.858 | 1 | 0.218 | 0.739 | **0** | 0.003 | 1 | 0.004 |
|  |  |  |  |  |  |  |  |  |  |  |
| **ZV** | 17 | **Na** | 4 | 5 | 9 | 14 | 6 | 6 | 1 | 2 |
|  |  | **Ar** | 4 | 4.979 | 8.763 | 13.26 | 5.754 | 5.882 | 1 | 1.989 |
|  |  | **Ho** | 0.625 | 0.765 | 0.941 | 0.941 | 0.529 | 0.941 | NA | 0.118 |
|  |  | **He** | 0.601 | 0.706 | 0.886 | 0.914 | 0.745 | 0.789 | NA | 0.114 |
|  |  | **p(HWE)** | 0.926 | 0.075 | 0.953 | 0.206 | 0.024 | 0.034 | NA | 1 |
|  |  |  |  |  |  |  |  |  |  |  |
| **CH** | 19 | **Na** | 5 | 9 | 12 | 16 | 7 | 2 | 1 | 1 |
|  |  | **Ar** | 4.81 | 8.329 | 10.49 | 14.89 | 6.736 | 2.000 | 1.000 | 1.000 |
|  |  | **Ho** | 0.833 | 0.895 | 0.789 | 0.895 | 0.632 | 0.947 | NA | NA |
|  |  | **He** | 0.711 | 0.862 | 0.851 | 0.940 | 0.718 | 0.512 | NA | NA |
|  |  | **p(HWE)** | 0.829 | **0** | 0.487 | 0.454 | 0.594 | 0.0003 | NA | NA |
|  |  |  |  |  |  |  |  |  |  |  |
| **XX** | 22 | **Na** | 4 | 9 | 9 | 14 | 7 | 4 | 2 | 2 |
|  |  | **Ar** | 3.904 | 8.017 | 7.938 | 12.15 | 6.543 | 3.904 | 1.904 | 2 |
|  |  | **Ho** | 0.636 | 0.955 | 0.727 | 0.864 | 0.682 | 0.952 | 0.091 | 0.364 |
|  |  | **He** | 0.630 | 0.849 | 0.817 | 0.909 | 0.744 | 0.617 | 0.089 | 0.304 |
|  |  | **p(HWE)** | 0.719 | 0.002 | 0.494 | 0.941 | 0.193 | **0** | 1 | 1 |
|  |  |  |  |  |  |  |  |  |  |  |
| **FD** | 29 | **Na** | 5 | 9 | 13 | 14 | 9 | 3 | 3 | 3 |
|  |  | **Ar** | 4.071 | 7.512 | 9.953 | 11.71 | 7.601 | 2.577 | 2.442 | 2.959 |
|  |  | **Ho** | 0.607 | 0.893 | 0.759 | 0.857 | 0.607 | 0.885 | 0.143 | 0.679 |
|  |  | **He** | 0.662 | 0.848 | 0.857 | 0.894 | 0.796 | 0.519 | 0.137 | 0.495 |
|  |  | **p(HWE)** | 0.103 | **0** | 0.003 | **0** | **0** | **0** | 1 | 0.058 |
|  |  |  |  |  |  |  |  |  |  |  |
| **BR** | 19 | **Na** | 5 | 8 | 11 | 14 | 6 | 2 | 1 | 2 |
|  |  | **Ar** | 4.954 | 7.783 | 10.59 | 13.12 | 5.539 | 2 | 1 | 1.999 |
|  |  | **Ho** | 0.684 | 0.944 | 0.944 | 0.947 | 0.474 | 0.944 | NA | 0.211 |
|  |  | **He** | 0.707 | 0.857 | 0.884 | 0.920 | 0.646 | 0.513 | NA | 0.193 |
|  |  | **p(HWE)** | 0.829 | **0** | 0.653 | 0.037 | 0.070 | 0.001 | NA | 1 |
|  |  |  |  |  |  |  |  |  |  |  |
| **TM** | 19 | **Na** | 4 | 9 | 9 | 10 | 9 | 3 | 1 | 1 |
|  |  | **Ar** | 4 | 8.629 | 8.866 | 9.956 | 8.105 | 2.96 | 1.000 | 1.000 |
|  |  | **Ho** | 0.467 | 0.842 | 0.895 | 0.588 | 0.632 | 0.737 | NA | NA |
|  |  | **He** | 0.494 | 0.838 | 0.872 | 0.907 | 0.677 | 0.512 | NA | NA |
|  |  | **p(HWE)** | 0.098 | 0.009 | 0.006 | **0** | 0.307 | 0.076 | NA | NA |
|  |  |  |  |  |  |  |  |  |  |  |
| **SB** | 30 | **Na** | 3 | 5 | 11 | 15 | 9 | 4 | 2 | 2 |
|  |  | **Ar** | 2.999 | 4.797 | 8.619 | 12.10 | 7.087 | 3 | 1.944 | 2 |
|  |  | **Ho** | 0.433 | 0.586 | 0.586 | 0.793 | 0.367 | 1 | 0.133 | 0.92 |
|  |  | **He** | 0.538 | 0.669 | 0.836 | 0.918 | 0.632 | 0.541 | 0.127 | 0.507 |
|  |  | **p(HWE)** | 0.279 | 0.487 | **0** | **0** | **0** | **0** | 1 | **0** |
|  |  |  |  |  |  |  |  |  |  |  |
| **PSJ** | 20 | **Na** | 3 | 9 | 13 | 13 | 9 | 4 | 3 | 2 |
|  |  | **Ar** | 3 | 8.223 | 11.72 | 12.74 | 8.525 | 3.831 | 2.5 | 2 |
|  |  | **Ho** | 0.722 | 0.950 | 0.850 | 0.563 | 0.632 | 0.889 | 0.1 | 0.222 |
|  |  | **He** | 0.667 | 0.829 | 0.919 | 0.909 | 0.844 | 0.589 | 0.099 | 0.203 |
|  |  | **p(HWE)** | 0.933 | 0.078 | 0.006 | **0** | 0.097 | 0.021 | 1 | 1 |
|  |  |  |  |  |  |  |  |  |  |  |
| **MB** | 21 | **Na** | 4 | 11 | 12 | 13 | 8 | 3 | 3 | 2 |
|  |  | **Ar** | 3.923 | 9.759 | 10.68 | 12.19 | 7.537 | 2.833 | 2.429 | 1.993 |
|  |  | **Ho** | 0.714 | 1 | 0.905 | 0.789 | 0.714 | 0.944 | 0.095 | 0.158 |
|  |  | **He** | 0.624 | 0.876 | 0.875 | 0.915 | 0.819 | 0.538 | 0.094 | 0.149 |
|  |  | **p(HWE)** | 0.956 | 0.007 | 0.841 | 0.135 | 0.055 | 0.001 | 1 | 1 |
|  |  |  |  |  |  |  |  |  |  |  |
| ***PHH*** | 43 | **Na** | 4 | 9 | 14 | 19 | 9 | 7 | 2 | 3 |
|  |  | **Ar** | 4 | 9 | 13.93 | 19 | 8.93 | 7 | 2 | 3 |
|  |  | **Ho** | 0.714 | 0.690 | 0.837 | 0.952 | 0.512 | 1 | 0.116 | 0.558 |
|  |  | **He** | 0.662 | 0.677 | 0.888 | 0.907 | 0.740 | 0.796 | 0.111 | 0.447 |
|  |  | **p(HWE)** | 0.758 | **0** | 0.093 | 0.144 | 0.004 | **0** | 1 | 0.150 |
|  |  |  |  |  |  |  |  |  |  |  |
| ***PHM*** | 53 | **Na** | 5 | 8 | 13 | 19 | 9 | 6 | 3 | 5 |
|  |  | **Ar** | 4.98 | 8 | 12.96 | 18.77 | 8.769 | 5.96 | 3 | 4.827 |
|  |  | **Ho** | 0.72 | 0.940 | 0.82 | 0.519 | 0.528 | 0.961 | 0.041 | 0.442 |
|  |  | **He** | 0.681 | 0.806 | 0.883 | 0.922 | 0.738 | 0.663 | 0.041 | 0.371 |
|  |  | **p(HWE)** | 0.480 | **0** | 0.008 | 0.011 | **0** | **0** | 1 | 0.141 |
|  |  |  |  |  |  |  |  |  |  |  |
| ***PHR*** | 175 | **Na** | 4.000 | 7.778 | 10.56 | 12.67 | 7.333 | 3.111 | 1.889 | 1.778 |
|  |  | **Ar** | 5 | 11.95 | 15.94 | 20.96 | 9.942 | 5.988 | 5.81 | 3 |
|  |  | **Ho** | 0.614 | 0.860 | 0.803 | 0.800 | 0.572 | 0.909 | 0.080 | 0.358 |
|  |  | **He** | 0.624 | 0.829 | 0.868 | 0.912 | 0.753 | 0.535 | 0.078 | 0.310 |
|  |  | **p(HWE)** | 0.707 | **0** | 0.052 | **0** | **0** | **0** | 1 | 0.059 |
|  |  |  |  |  |  |  |  |  |  |  |
| **PIC** |  |  | 0.605 | 0.809 | 0.872 | 0.917 | 0.730 | 0.550 | 0.072 | 0.314 |
| **% Genotyping error** |  |  | 0 | 0 | 0 | 0 | 0 | 0 | 0 | 0 |

Table S3 B. Microsatellite summary statistics by population and locus for the Delghandi et al. (2015) primer set. N = number of individuals, Na = number of alleles, Ar = allelic richness, Ho = observed heterozygosity, He = expected heterozygosity, p(HWE) = probability of deviation from HWE, PHH = *P. h. homarus*, PHM = *P. h. megasculptus*, PHR = *P. h. rubellus*, PIC = polymorphic information content.

| **Location/ Subspecies** | **N** |  | **G01** | **G03** | **G21** | **G22** | **G25** | **G27** | **G30** | **G32** | **G35** | **G36** | **G42** | **G53** | **G58** |
| --- | --- | --- | --- | --- | --- | --- | --- | --- | --- | --- | --- | --- | --- | --- | --- |
| **All** | 271 | **Na** | 5.333 | 4.000 | 4.167 | 5.833 | 8.667 | 2.250 | 4.500 | 3.583 | 10.667 | 9.083 | 2.083 | 7.917 | 1.917 |
|  |  | **Ar** | 5.187 | 4.124 | 4.525 | 5.723 | 8.227 | 2.514 | 4.462 | 3.881 | 10.37 | 9.377 | 2.161 | 7.744 | 2 |
|  |  | **Ho** | 0.668 | 0.556 | 0.309 | 0.742 | 0.733 | 0.157 | 0.490 | 0.194 | 0.874 | 0.950 | 0.989 | 0.735 | 0.299 |
|  |  | **He** | 0.712 | 0.516 | 0.657 | 0.669 | 0.849 | 0.196 | 0.538 | 0.551 | 0.871 | 0.857 | 0.506 | 0.806 | 0.369 |
|  |  | **p(HWE)** | 0.025 | **0** | **0** | **0** | 0.118 | **0** | 0.283 | **0** | 0.379 | **0** | **0** | **0** | 0.004 |
|  |  |  |  |  |  |  |  |  |  |  |  |  |  |  |  |
| **OM** | 29 | **Na** | 5 | 6 | 5 | 6 | 9 | 3 | 5 | 4 | 14 | 16 | 3 | 12 | 2 |
|  |  | **Ar** | 4.665 | 5.021 | 4.542 | 5.687 | 8.593 | 2.906 | 4.772 | 3.495 | 10.59 | 12.99 | 2.894 | 9.12 | 1.517 |
|  |  | **Ho** | 0.689 | 0.689 | 0.655 | 0.821 | 1 | 0.357 | 0.607 | 0.379 | 0.828 | 1 | 1 | 0.828 | 0.034 |
|  |  | **He** | 0.719 | 0.617 | 0.619 | 0.712 | 0.874 | 0.382 | 0.570 | 0.426 | 0.883 | 0.919 | 0.556 | 0.857 | 0.034 |
|  |  | **p(HWE)** | 0.511 | 0.385 | 0.208 | 0.241 | 0.929 | 0.017 | 0.568 | 0.016 | 0.062 | 0.021 | **0** | 0.443 | 1 |
|  |  |  |  |  |  |  |  |  |  |  |  |  |  |  |  |
| **YEM** | 24 | **Na** | 5 | 4 | 4 | 7 | 9 | 2 | 6 | 4 | 12 | 13 | 2 | 11 | 1 |
|  |  | **Ar** | 4.304 | 3.979 | 3.625 | 6.072 | 8.378 | 1.989 | 4.979 | 3.884 | 10.65 | 11.73 | 2 | 9.798 | 1 |
|  |  | **Ho** | 0.652 | 0.75 | 0.375 | 0.565 | 0.609 | 0 | 0.458 | 0.391 | 1 | 0.636 | 1 | 0.909 | NA |
|  |  | **He** | 0.647 | 0.602 | 0.576 | 0.673 | 0.816 | 0.162 | 0.569 | 0.658 | 0.884 | 0.891 | 0.511 | 0.879 | NA |
|  |  | **p(HWE)** | 0.699 | 0.339 | 0.006 | 0.249 | 0.183 | 0.001 | 0.646 | 0.009 | 0.985 | **0** | **0** | 0.546 | NA |
|  |  |  |  |  |  |  |  |  |  |  |  |  |  |  |  |
| **KEN** | 22 | **Na** | 4 | 2 | 4 | 4 | 10 | 2 | 4 | 5 | 7 | 5 | 2 | 7 | 2 |
|  |  | **Ar** | 3.364 | 2 | 3.902 | 3.586 | 8.859 | 1.682 | 3.694 | 4.896 | 6.548 | 4.965 | 2 | 6.351 | 1.999 |
|  |  | **Ho** | 0.5 | 0.364 | 0.182 | 0.864 | 0.818 | 0.045 | 0.190 | 0.364 | 0.727 | 0.955 | 1 | 0.762 | 0.238 |
|  |  | **He** | 0.470 | 0.304 | 0.625 | 0.555 | 0.818 | 0.045 | 0.372 | 0.719 | 0.696 | 0.737 | 0.512 | 0.777 | 0.215 |
|  |  | **p(HWE)** | 0.664 | 1 | **0** | 0.001 | 0.406 | 1 | 0.011 | **0** | 0.776 | 0.026 | **0** | 0.242 | 1 |
|  |  |  |  |  |  |  |  |  |  |  |  |  |  |  |  |
| **ZV** | 17 | **Na** | 5 | 4 | 4 | 4 | 7 | 2 | 3 | 4 | 8 | 7 | 2 | 5 | 2 |
|  |  | **Ar** | 4.872 | 3.882 | 3.882 | 3.765 | 6.743 | 2 | 2.765 | 3.999 | 7.529 | 6.871 | 2.000 | 4.989 | 2.000 |
|  |  | **Ho** | 0.529 | 0.588 | 0.118 | 1 | 0.529 | 0.529 | 0.118 | 0.235 | 0.647 | 1 | 1 | 0.647 | 0.235 |
|  |  | **He** | 0.611 | 0.506 | 0.620 | 0.572 | 0.750 | 0.401 | 0.116 | 0.672 | 0.693 | 0.834 | 0.515 | 0.739 | 0.214 |
|  |  | **p(HWE)** | 0.653 | 1 | **0** | **0** | 0.031 | 0.288 | 1 | 0.0001 | 0.161 | 0.114 | **0** | 0.392 | 1 |
|  |  |  |  |  |  |  |  |  |  |  |  |  |  |  |  |
| **CH** | 19 | **Na** | 6 | 3 | 4 | 7 | 8 | 2 | 3 | 3 | 10 | 8 | 2 | 5 | 2 |
|  |  | **Ar** | 5.92 | 3.000 | 3.833 | 6.619 | 7.497 | 1.976 | 3 | 2.999 | 9.742 | 7.86 | 2.000 | 4.833 | 2.000 |
|  |  | **Ho** | 0.737 | 0.278 | 0.111 | 0.611 | 0.611 | 0.111 | 0.556 | 0.053 | 0.824 | 1 | 1 | 0.833 | 0.556 |
|  |  | **He** | 0.787 | 0.417 | 0.576 | 0.705 | 0.779 | 0.108 | 0.603 | 0.605 | 0.893 | 0.852 | 0.514 | 0.779 | 0.508 |
|  |  | **p(HWE)** | 0.812 | 0.01 | **0** | 0.028 | 0.296 | 1 | 0.340 | **0** | 0.373 | 0.088 | **0** | 0.945 | 1 |
|  |  |  |  |  |  |  |  |  |  |  |  |  |  |  |  |
| **XX** | 22 | **Na** | 5 | 4 | 5 | 7 | 11 | 3 | 7 | 4 | 11 | 9 | 2 | 6 | 2 |
|  |  | **Ar** | 4.938 | 3.902 | 4.364 | 6.171 | 9.305 | 2.904 | 6.238 | 3.68 | 9.478 | 8.169 | 2 | 5.336 | 2.000 |
|  |  | **Ho** | 0.591 | 0.500 | 0.136 | 0.864 | 0.591 | 0.364 | 0.591 | 0.227 | 0.864 | 1 | 0.955 | 0.773 | 0.318 |
|  |  | **He** | 0.648 | 0.481 | 0.651 | 0.719 | 0.859 | 0.376 | 0.626 | 0.560 | 0.808 | 0.848 | 0.511 | 0.729 | 0.426 |
|  |  | **p(HWE)** | 0.439 | 0.513 | **0** | 0.007 | 0.011 | 0.039 | 0.520 | 0.0005 | 0.072 | **0** | **0** | 0.919 | 0.318 |
|  |  |  |  |  |  |  |  |  |  |  |  |  |  |  |  |
| **FD** | 29 | **Na** | 6 | 4 | 5 | 7 | 9 | 3 | 5 | 4 | 14 | 10 | 2 | 9 | 2 |
|  |  | **Ar** | 5.577 | 3.948 | 4.772 | 5.472 | 8.392 | 2.885 | 4.325 | 3.439 | 11.30 | 8.535 | 2 | 7.532 | 2 |
|  |  | **Ho** | 0.679 | 0.586 | 0.357 | 0.778 | 0.852 | 0.111 | 0.607 | 0.214 | 0.926 | 1 | 1 | 0.741 | 0.241 |
|  |  | **He** | 0.765 | 0.554 | 0.658 | 0.674 | 0.855 | 0.238 | 0.684 | 0.342 | 0.904 | 0.796 | 0.509 | 0.819 | 0.390 |
|  |  | **p(HWE)** | 0.113 | 0.170 | 0.001 | **0** | 0.423 | 0.002 | 0.191 | 0.034 | 0.440 | 0.124 | **0** | 0.633 | 0.055 |
|  |  |  |  |  |  |  |  |  |  |  |  |  |  |  |  |
| **BR** | 19 | **Na** | 5 | 4 | 3 | 5 | 9 | 2 | 4 | 3 | 10 | 9 | 2 | 8 | 2 |
|  |  | **Ar** | 4.749 | 3.973 | 3 | 4.667 | 8.637 | 1.997 | 3.81 | 2.789 | 9.59 | 8.738 | 2 | 7.151 | 2 |
|  |  | **Ho** | 0.421 | 0.667 | 0.188 | 0.667 | 0.889 | 0.167 | 0.333 | 0.105 | 1 | 1 | 1 | 0.684 | 0.667 |
|  |  | **He** | 0.669 | 0.522 | 0.522 | 0.662 | 0.865 | 0.157 | 0.383 | 0.459 | 0.876 | 0.827 | 0.514 | 0.767 | 0.508 |
|  |  | **p(HWE)** | 0.025 | 0.647 | 0.0005 | 0.009 | 0.854 | 1 | 0.655 | **0** | 0.941 | 0.049 | 0.0005 | 0.070 | 0.334 |
|  |  |  |  |  |  |  |  |  |  |  |  |  |  |  |  |
| **TM** | 19 | **Na** | 5 | 4 | 4 | 5 | 7 | 2 | 4 | 2 | 10 | 6 | 2 | 8 | 2 |
|  |  | **Ar** | 4.954 | 3.947 | 3.789 | 4.789 | 6.742 | 1.96 | 3.959 | 2 | 9.458 | 5.539 | 2 | 7.702 | 2 |
|  |  | **Ho** | 0.895 | 0.421 | 0.368 | 0.684 | 0.526 | 0 | 0.684 | 0 | 0.947 | 1 | 1 | 0.474 | 0.421 |
|  |  | **He** | 0.724 | 0.371 | 0.644 | 0.650 | 0.812 | 0.102 | 0.558 | 0.273 | 0.883 | 0.728 | 0.513 | 0.811 | 0.478 |
|  |  | **p(HWE)** | 0.587 | 1 | 0.009 | 0.233 | 0.001 | 0.025 | 0.934 | **0** | 0.198 | 0.025 | **0** | **0** | 0.643 |
|  |  |  |  |  |  |  |  |  |  |  |  |  |  |  |  |
| **SB** | 30 | **Na** | 7 | 4 | 5 | 8 | 9 | 2 | 4 | 3 | 12 | 9 | 2 | 9 | 2 |
|  |  | **Ar** | 5.88 | 3.535 | 4.892 | 6.085 | 7.436 | 1.517 | 3.872 | 2.517 | 10.15 | 7.615 | 2 | 7.451 | 2 |
|  |  | **Ho** | 0.7 | 0.571 | 0.357 | 0.759 | 0.655 | 0.034 | 0.517 | 0.034 | 0.867 | 1 | 0.964 | 0.759 | 0.321 |
|  |  | **He** | 0.771 | 0.537 | 0.718 | 0.642 | 0.842 | 0.034 | 0.556 | 0.416 | 0.873 | 0.793 | 0.508 | 0.797 | 0.431 |
|  |  | **p(HWE)** | 0.683 | 0.066 | **0** | 0.101 | 0.176 | 1 | 0.628 | **0** | 0.929 | 0.077 | **0** | 0.067 | 0.229 |
|  |  |  |  |  |  |  |  |  |  |  |  |  |  |  |  |
| **PSJ** | 20 | **Na** | 5 | 5 | 3 | 4 | 7 | 2 | 4 | 3 | 10 | 9 | 2 | 7 | 2 |
|  |  | **Ar** | 4.783 | 4.635 | 3 | 3.994 | 6.996 | 1.789 | 3.748 | 2.75 | 9.733 | 8.615 | 2 | 6.873 | 2 |
|  |  | **Ho** | 0.789 | 0.55 | 0.389 | 0.389 | 0.875 | 0.053 | 0.600 | 0.05 | 1 | 0.882 | 1 | 0.500 | 0.400 |
|  |  | **He** | 0.739 | 0.486 | 0.608 | 0.579 | 0.855 | 0.053 | 0.506 | 0.537 | 0.891 | 0.752 | 0.514 | 0.782 | 0.385 |
|  |  | **p(HWE)** | 0.114 | 0.372 | 0.012 | 0.032 | 0.439 | 1 | 0.882 | **0** | 1 | 0.963 | 0.0001 | 0.026 | 1 |
|  |  |  |  |  |  |  |  |  |  |  |  |  |  |  |  |
| **MB** | 21 | **Na** | 6 | 4 | 4 | 6 | 9 | 2 | 5 | 4 | 10 | 8 | 2 | 8 | 2 |
|  |  | **Ar** | 5.766 | 3.998 | 3.831 | 5.539 | 8.536 | 1.993 | 4.539 | 3.638 | 9.251 | 6.989 | 2 | 7.124 | 2 |
|  |  | **Ho** | 0.809 | 0.600 | 0.222 | 0.842 | 0.737 | 0.158 | 0.474 | 0.190 | 0.857 | 0.905 | 0.947 | 0.762 | 0.421 |
|  |  | **He** | 0.700 | 0.622 | 0.537 | 0.706 | 0.859 | 0.149 | 0.496 | 0.475 | 0.877 | 0.746 | 0.512 | 0.819 | 0.478 |
|  |  | **p(HWE)** | 0.799 | 0.231 | **0** | 0.091 | 0.162 | 1 | 0.098 | 0.001 | 0.608 | 0.266 | 0.0005 | 0.205 | 0.654 |
|  |  |  |  |  |  |  |  |  |  |  |  |  |  |  |  |
| ***PHH*** | 43 | **Na** | 5 | 4 | 4 | 5 | 11 | 2 | 5 | 5 | 9 | 9 | 2 | 8 | 2 |
|  |  | **Ar** | 5 | 3.977 | 4 | 4.999 | 10.95 | 2 | 5 | 5 | 8.953 | 8.93 | 2.000 | 8.000 | 2.000 |
|  |  | **Ho** | 0.488 | 0.442 | 0.139 | 0.884 | 0.628 | 0.209 | 0.214 | 0.372 | 0.628 | 0.977 | 0.977 | 0.762 | 0.190 |
|  |  | **He** | 0.553 | 0.386 | 0.596 | 0.582 | 0.758 | 0.189 | 0.336 | 0.721 | 0.654 | 0.776 | 0.506 | 0.740 | 0.248 |
|  |  | **p(HWE)** | 0.507 | 0.806 | **0** | **0** | 0.115 | 1 | 0.017 | **0** | 0.192 | **0** | **0** | 0.376 | 0.178 |
|  |  |  |  |  |  |  |  |  |  |  |  |  |  |  |  |
| ***PHM*** | 53 | **Na** | 5 | 6 | 5 | 7 | 9 | 3 | 7 | 4 | 16 | 16 | 3 | 12 | 2 |
|  |  | **Ar** | 5 | 5.849 | 4.995 | 6.961 | 9 | 3 | 6.939 | 4 | 15.84 | 15.96 | 3 | 11.96 | 1.925 |
|  |  | **Ho** | 0.673 | 0.717 | 0.528 | 0.706 | 0.824 | 0.196 | 0.538 | 0.385 | 0.902 | 0.843 | 1 | 0.863 | 0.019 |
|  |  | **He** | 0.688 | 0.608 | 0.595 | 0.702 | 0.856 | 0.294 | 0.568 | 0.545 | 0.881 | 0.908 | 0.532 | 0.869 | 0.019 |
|  |  | **p(HWE)** | 0.296 | 0.017 | 0.031 | 0.325 | 0.632 | 0.001 | 0.867 | **0** | 0.685 | **0** | **0** | 0.219 | 1 |
|  |  |  |  |  |  |  |  |  |  |  |  |  |  |  |  |
| ***PHR*** | 175 | **Na** | 5.444 | 4.000 | 4.000 | 5.778 | 8.333 | 2.222 | 4.222 | 2.667 | 10.22 | 8.000 | 2.000 | 7.222 | 2.000 |
|  |  | **Ar** | 6.942 | 4.998 | 5 | 10.95 | 12.98 | 3 | 7.927 | 4.942 | 17.94 | 11.96 | 2 | 11.98 | 2 |
|  |  | **Ho** | 0.711 | 0.535 | 0.282 | 0.717 | 0.732 | 0.132 | 0.544 | 0.092 | 0.929 | 0.976 | 0.988 | 0.689 | 0.414 |
|  |  | **He** | 0.735 | 0.514 | 0.641 | 0.668 | 0.855 | 0.167 | 0.564 | 0.446 | 0.877 | 0.788 | 0.501 | 0.793 | 0.452 |
|  |  | **p(HWE)** | 0.086 | 0.001 | **0** | **0** | 0.097 | **0** | 0.406 | **0** | 0.675 | **0** | **0** | **0** | 0.336 |
|  |  |  |  |  |  |  |  |  |  |  |  |  |  |  |  |
| **PIC** |  |  | 0.664 | 0.484 | 0.589 | 0.629 | 0.829 | 0.185 | 0.500 | 0.502 | 0.857 | 0.840 | 0.386 | 0.782 | 0.302 |
| **% Genotyping error** |  |  | 1.9 | 0 | 0 | 0 | 0 | 0 | 0 | 0 | 0 | 3.7 | 0 | 9.2 | 0 |

Table S4. Pairwise F_ST_ values for the microsatellite *P. homarus* dataset. Values in bold indicate significance.

|  | 1 | 2 | 3 | 4 | 5 | 6 | 7 | 8 | 9 | 10 | 11 | 12 |
| --- | --- | --- | --- | --- | --- | --- | --- | --- | --- | --- | --- | --- |
| 1. OM | * |  |  |  |  |  |  |  |  |  |  |  |
| 2. YEM | **0.008** | * |  |  |  |  |  |  |  |  |  |  |
| 3. KEN | **0.082** | **0.072** | * |  |  |  |  |  |  |  |  |  |
| 4. ZV | **0.056** | **0.064** | **0.016** | * |  |  |  |  |  |  |  |  |
| 5. CH | **0.045** | **0.050** | **0.090** | **0.066** | * |  |  |  |  |  |  |  |
| 6. XX | **0.033** | **0.037** | **0.051** | **0.015** | **0.019** | * |  |  |  |  |  |  |
| 7. FD | **0.031** | **0.036** | **0.077** | **0.055** | **0.029** | 0.004 | * |  |  |  |  |  |
| 8. BR | **0.046** | **0.060** | **0.093** | **0.052** | **0.015** | 0.005 | **0.010** | * |  |  |  |  |
| 9. TM | **0.055** | **0.059** | **0.094** | **0.052** | **0.023** | **0.010** | **0.020** | **0.011** | * |  |  |  |
| 10. SB | **0.064** | **0.060** | **0.065** | **0.055** | **0.037** | **0.016** | **0.015** | **0.025** | **0.023** | * |  |  |
| 11. PSJ | **0.035** | **0.038** | **0.075** | **0.051** | **0.014** | 0.007 | **0.012** | 0.006 | **0.015** | **0.025** | * |  |
| 12. MB | **0.038** | **0.044** | **0.075** | **0.039** | **0.024** | -0.007 | 0.002 | 0.002 | 0.004 | **0.020** | **0.008** | * |


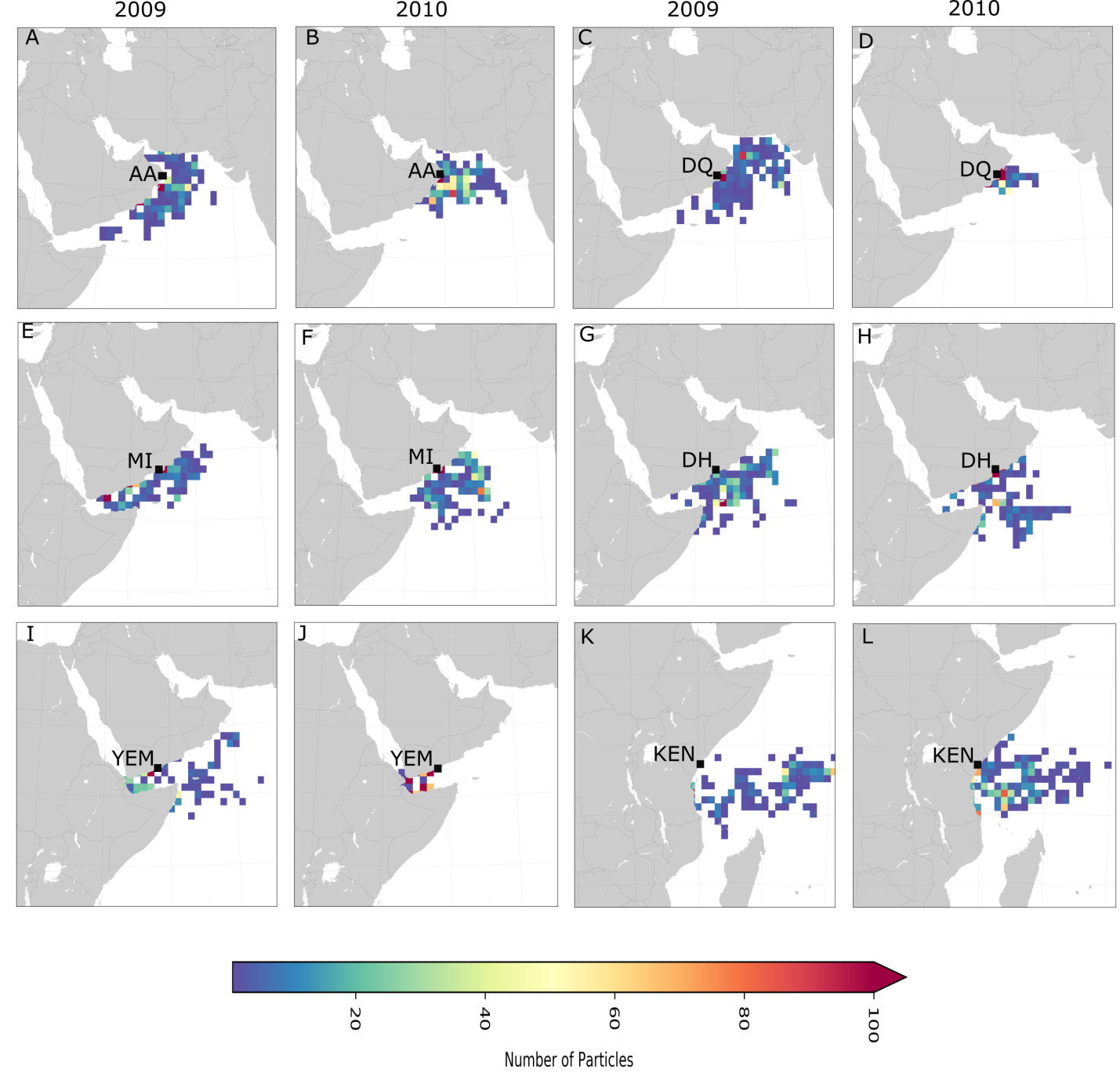


Figure S1. Particle density plots of the Lagrangian trajectories on a 1°x1°spatial grid for the northern sites for January 2009 and 2010. AA = Al Ashkharah, DQ = Duqm, MI = Mirbat, DH = Dhalkut, YEM = Yemen, KEN = Kenya.


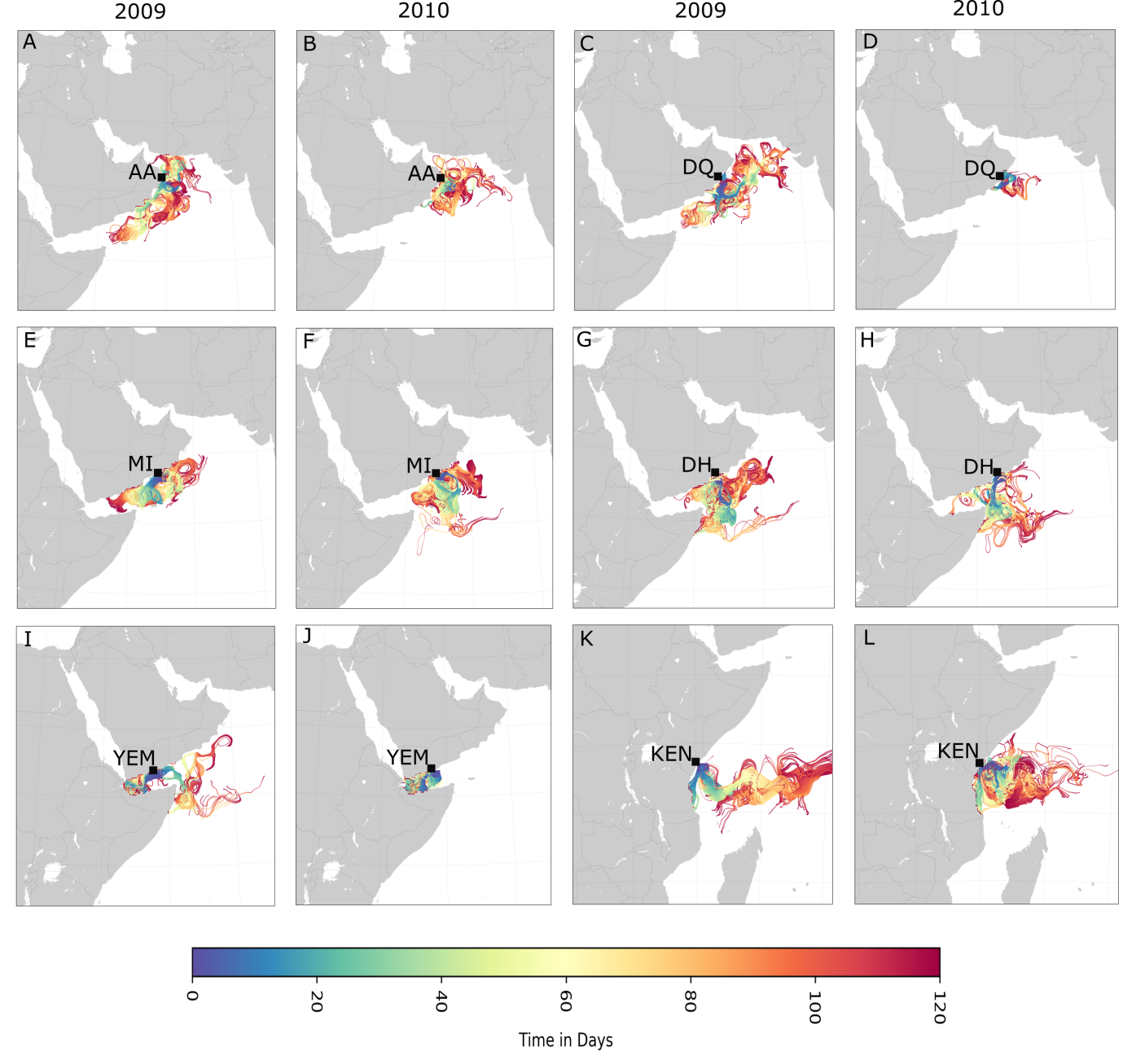


Figure S2. Particle dispersal trajectories for the northern sites for January 2009 and 2010. AA = Al Ashkharah, DQ = Duqm, MI = Mirbat, DH = Dhalkut, YEM = Yemen, KEN = Kenya.


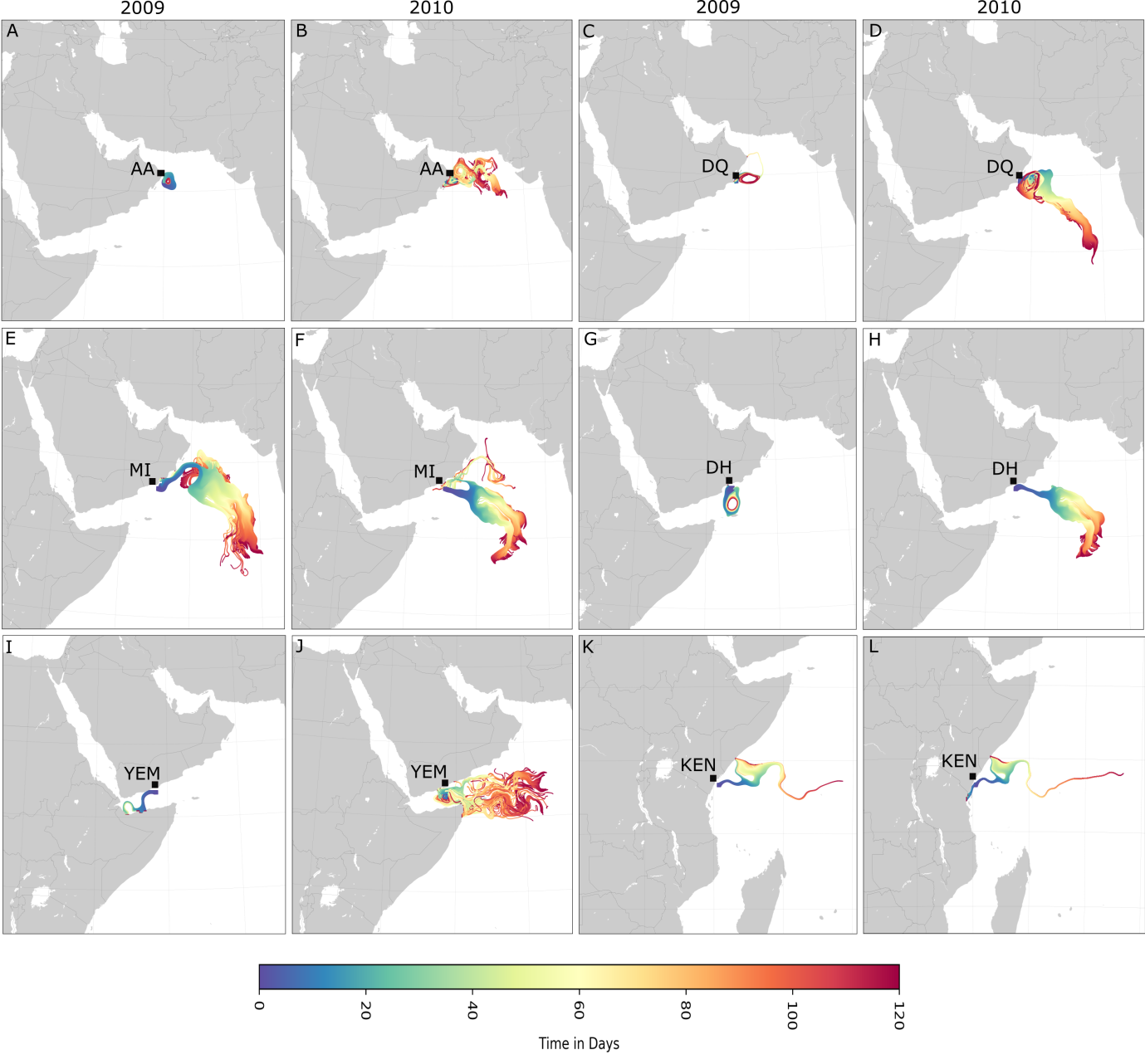


Figure S3. Particle dispersal trajectories for the northern sites for June 2009 and 2010. AA = Al Ashkharah, DQ = Duqm, MI = Mirbat, DH = Dhalkut, YEM = Yemen, KEN = Kenya.


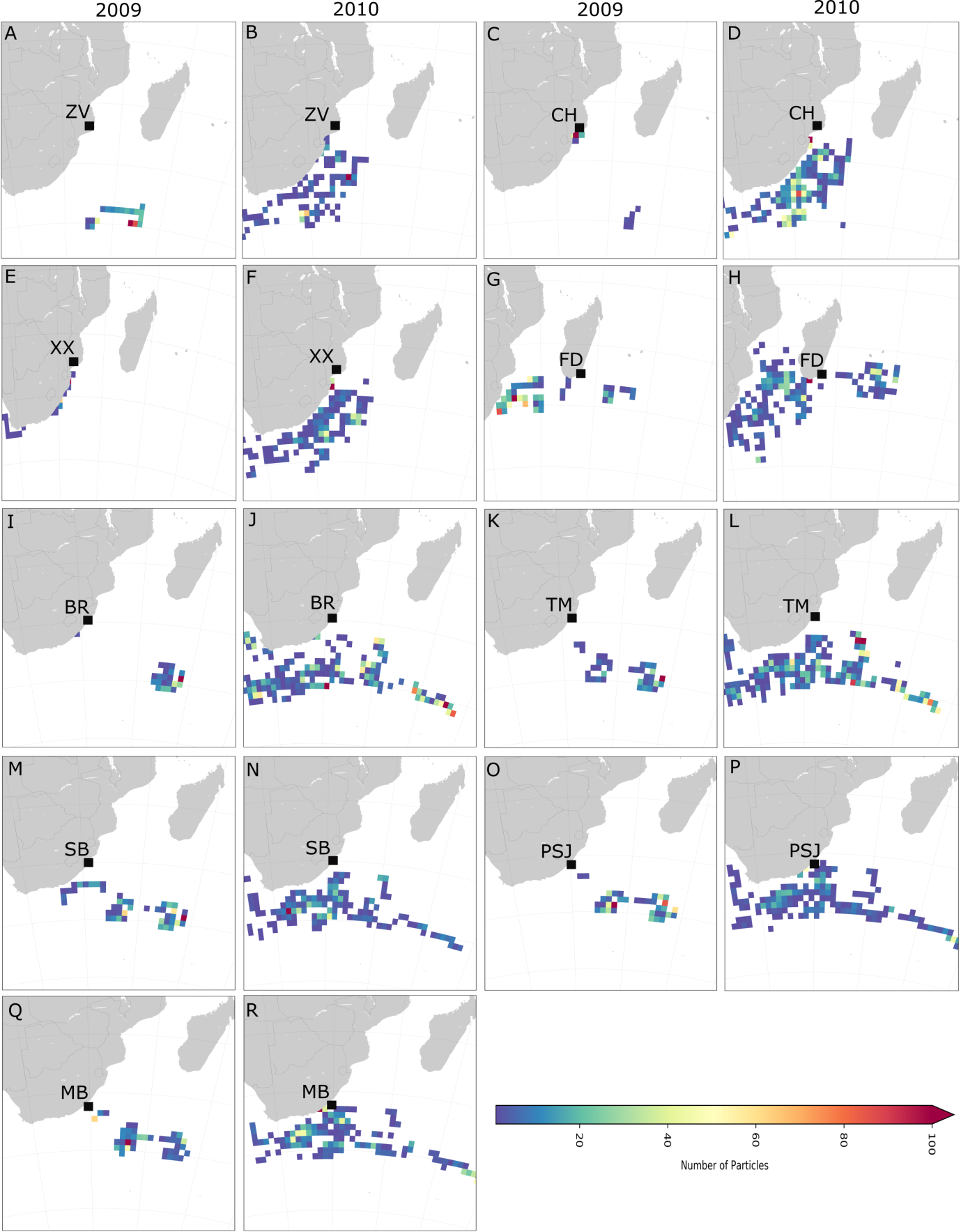


Figure S4. Particle density plots of the Lagrangian trajectories on a 1°x1°spatial grid for the southern sites for June 2009 and 2010. ZV = Zavora, CH = Chidenguele, XX = Xai Xai, TM = Tinley Manor, BR = Blood Reef, SB = Scottburgh, PSJ = Port St Johns, MB = Mdumbi.


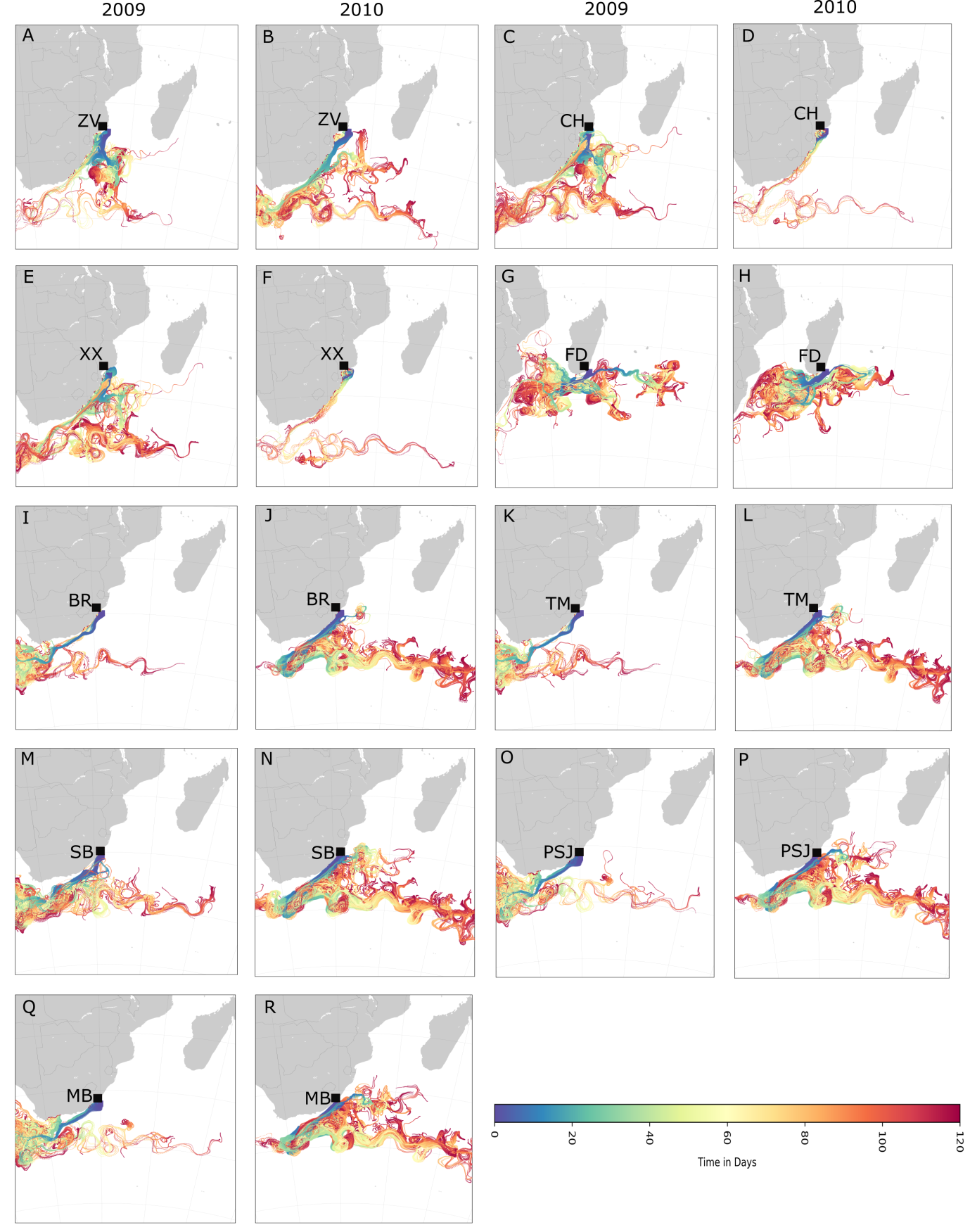
Figure S5. Particle dispersal trajectories for the southern sites for January 2009 and 2010. ZV = Zavora, CH = Chidenguele, XX = Xai Xai, TM = Tinley Manor, BR = Blood Reef, SB = Scottburgh, PSJ = Port St Johns, MB = Mdumbi.


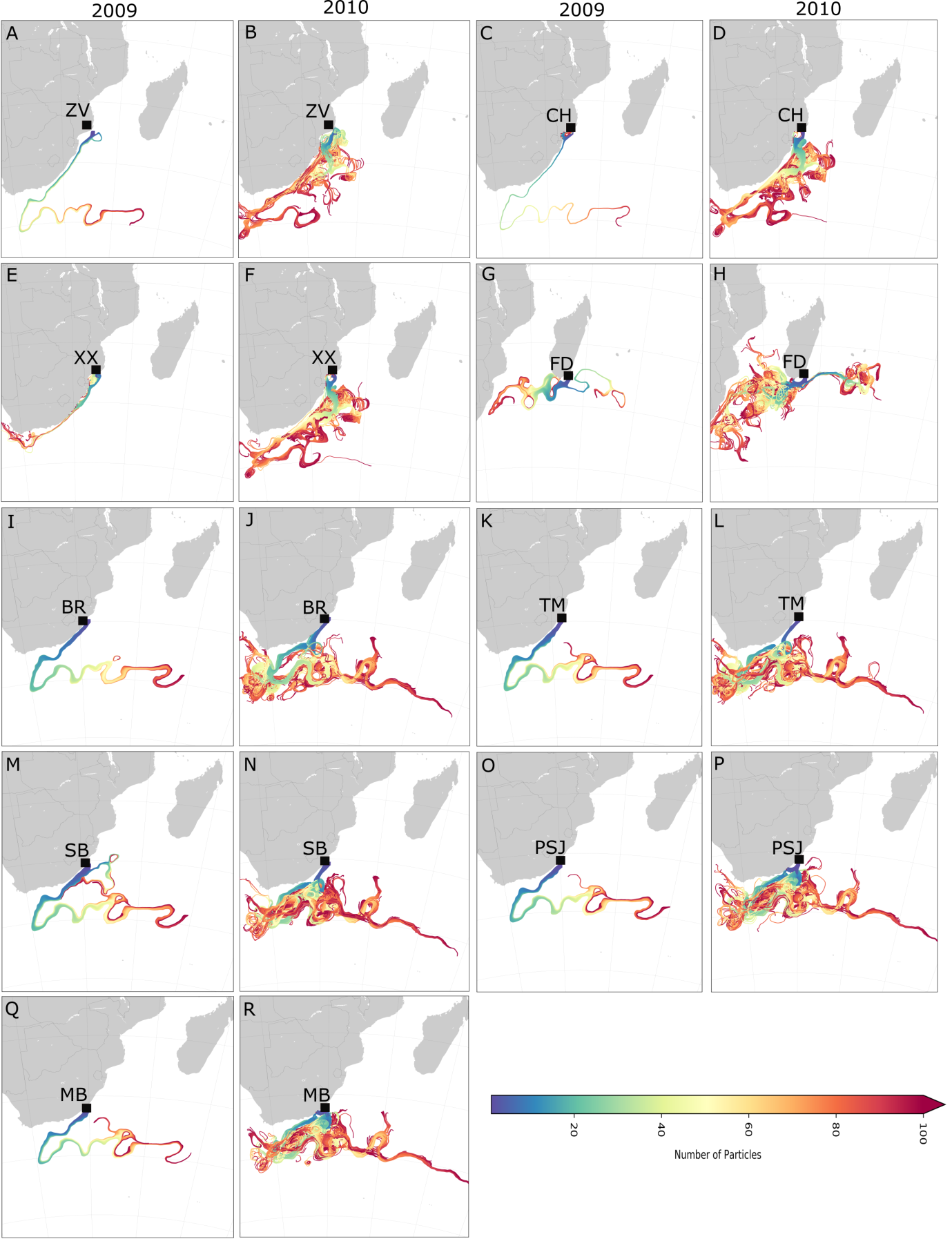


Figure S6. Particle dispersal trajectories for the southern sites for June 2009 and 2010. ZV = Zavora, CH = Chidenguele, XX = Xai Xai, TM = Tinley Manor, BR = Blood Reef, SB = Scottburgh, PSJ = Port St Johns, MB = Mdumbi.
